# Orphan Three-Finger Toxins from Snake Venoms: Unexplored Library of Novel Biological Ligands with Potential New Structures and Functions

**DOI:** 10.3390/ijms26188792

**Published:** 2025-09-09

**Authors:** Cho Yeow Koh, R. Manjunatha Kini

**Affiliations:** 1 Department of Medicine, Yong Loo Lin School of Medicine, National University of Singapore, Singapore 117599, Singapore; 2 Department of Pharmacology, Yong Loo Lin School of Medicine, National University of Singapore, Singapore 117600, Singapore; 3 Department of Biological Sciences, Faculty of Science, National University of Singapore, Singapore 117558, Singapore; 4 Department of Biochemistry, Medical College of Virginia, Virginia Commonwealth University, Richmond, VA 23298, USA

**Keywords:** neurotoxins, disulfide, novel pharmacophores, inhibitors, protein scaffold, toxin library

## Abstract

Three-finger toxins (3FTxs) from snake venom are the most abundant toxin family of mini non-enzymatic proteins, comprising 40–70% of the venom proteome. Despite their common three-finger structural scaffold, 3FTxs exhibit diverse pharmacological functions. Other than neurotoxins, they also include analgesic acid-sensing ion channel blockers, sodium and potassium channel modulators, integrin- and G-protein-coupled-receptor-targeting ligands, and gamma-aminobutyric acid type A receptor modulators that collectively span pain, cardiovascular, oncologic, and neurologic indications. However, in this fast-growing 3FTx family, there are several hundred 3FTxs whose functions have not yet been determined. Here, we systematically analyzed over 550 amino acid sequences of 3FTxs. Based on their structural features, we have classified them into more than 150 distinct subgroups. This updated information on this novel 3FTx toolkit will provide an unexplored library of investigational ligands and pharmacophores with potential therapeutic and diagnostic leads, as well as research tools. Thus, this review will provide new impetus in toxin research and pave the way for the design of potent, selective ligands for new sets of target receptors, ion channels, and enzymes.

## 1. Introduction

**Figure 1:**
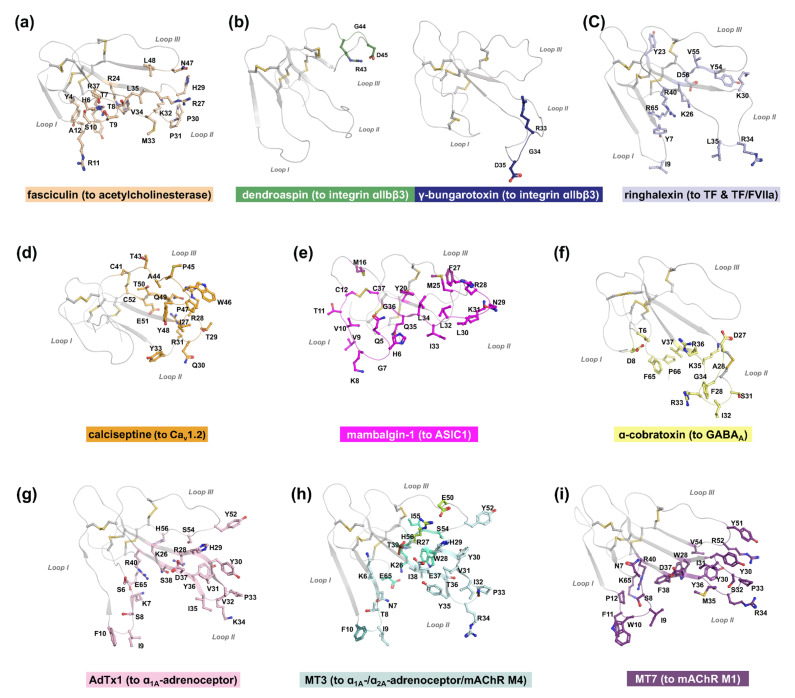
3FTxs known to bind to receptors/ion channels/enzymes other than nAChR. All 3FTxs are structurally aligned to depict the same orientation. Residues involved in binding to their respective targets are shown in ball and stick models. (**a**) Fasciculin-2, residues (colored wheat) within 5Å radius of acetylcholinesterase were identified from PDB entry 4EY8. (**b**) Both dendroaspin (PDB: 1DRS, green) and γ-bungarotoxin (PDB: 1MR6, deep blue) are shown. Despite the different locations of RGD sequence (Loop III and Loop II for dendroaspin and γ-bungarotoxin, respectively), both bind to integrin αIIbβ3. (**c**) Ringhalexin (PDB: 4ZQY), residues (colored lavender) predicted to bind to TF and TF/VIIa in docking study were identified. (**d**) Calciseptine, residues (colored orange) within 5Å radius of Ca_v_1.2 were identified from PDB entry 8WE8. (**e**) Mambalgin-1, residues (colored magenta) within 5Å radius of ASIC1 were identified from PDB entry 7CFT. (**f**) α-cobratoxin, residues (colored yellow) within 5Å radius of GABA_A_ were identified from PDB entry 7PC0. (**g**) AdTx1 (ρ-Da1a), residues (colored light pink) within 5Å radius of α_1A_-adrenoceptor were identified from PDB entry 8HN1. (**h**) High-resolution structures of MT3 in complex with α_1A_-adrenoceptor (PDB: 9IQV), α_2A_-adrenoceptor (PDB: 9IQR), and mAChR M4 (PDB: 9IQS) are available. Residues within 5Å radius of all three receptors are colored light blue. Residue Phe10 (colored teal) is within 5Å radius of the two adrenoceptors but not mAChR M4. Lys26, Trp28, Ser54, Ile55, His56, and Glu65 (colored green-cyan) bind only to α_1A_-adrenoceptor. Arg27 and Glu50 (colored lemon) bind only to α_2A_-adrenoceptor. Thr39 (colored brown) binds only to mAChR M4. (**i**) MT7, residues (colored violet-purple) within 5Å radius of acetylcholinesterase were identified from PDB entry 6WJC.

Snake venom toxins belong to a small number of superfamilies of enzymes and non-enzymatic proteins. One of the well-recognized, abundant families of non-enzymatic venom proteins is that of three-finger toxins (3FTxs) [1,2,3]. Members of this multigene family contain 57 to 82 amino acid residues. They have a similar compact protein folding scaffold, built by three β-stranded loops extending from a central core stabilized by four conserved disulfide bridges, resulting in an uncanny resemblance to three fingers stretched from the palm (the central core), hence the name “three-finger” toxin. 3FTxs are rich in disulfide bonds. In addition to the four conserved disulfide bridges in the central core (or palm), some 3FTx types occasionally have an extra fifth disulfide bond, located either in their central loop II (e.g., long-chain α-neurotoxins and κ-neurotoxins) or in the N-terminal loop I (e.g., non-conventional neurotoxins) [4]. Loops I and II are topologically interlocked in the central core, and the overlapped segment is described as the “head” here. Meanwhile, loop III is linked to loop II via a short segment of residues, described as the “gap” here. Interestingly, despite their common three-finger fold, these polypeptides greatly differ in their biological activities [1,2,3]. Their diverse biological activities involve selective interactions with a wide array of receptors and ion channels (Figure 1). For example, neurotoxins antagonize nicotinic acetylcholine receptors (nAChRs) [5]; aminergic or muscarinic toxins interact with biogenic amine receptors and muscarinic acetylcholine receptors (mAChRs) [6,7,8,9,10]; calciseptine interact with Ca^2+^ channels [11,12]; mambalgins block acid-sensing ion channels [13,14]; and micrurotoxin and α-cobratoxin interact with gamma-aminobutyric acid type A (GABA_A_) receptors [12,15]. Other selective interactions include dendroaspin and γ-bungarotoxin interacting with integrins [16,17,18], hemextin and ringhalexin inhibiting factor VIIa [19,20], and fasciculin inhibiting acetylcholinesterases [21,22]. In addition to these selective activities, some 3FTxs exhibit non-selective interaction-mediated cell lysis. For example, cardiotoxins or cytotoxins interact with phospholipids and cell membranes [23]. In addition, novel and unexpected biological activities are seen in some 3FTxs, such as cardiotoxin-I interaction with interleukin or insulin receptors [24] and the 3FTx-activated motility of sperm exhibited by actiflagelin [25].

The scope for discovering new animal toxins with novel pharmacological functions that can be utilized as investigational ligand tools in research and as therapeutic leads for drug discovery has broadened [26,27,28]. In addition to classical α-three-finger neurotoxins (e.g., short-chain and long-chain types), several new classes of neurotoxins such as non-covalent homodimers (haditoxin and fulditoxin were added to κ-bungarotoxins) [29,30], disulfide-bound covalent hetero- and homodimers (heterodimer of α-cobratoxin with various cytotoxins, homodimer of α-cobratoxin and irditoxin) [31,32,33], non-conventional α-neurotoxins (e.g., candoxin) [34], or toxins with unique sequences (Ω-neurotoxins) [35] are characterized. Their intended interests notwithstanding, the ability of 3FTxs to affect a diverse range of molecular targets with a few subtle changes in the functional sites and structure makes them an exciting group of toxins.

In this fast-growing 3FTx family, there are numerous toxins whose functions are not yet determined. In 2003, we performed phylogenetic analysis of 3FTxs and described the diversity of family members [2]. A significant proportion of these 3FTxs belonged to clades with unknown functions. Therefore, these 3FTxs were grouped into 20 different orphan clades (I–XX) with individual toxins showing 75% sequence similarity. In the last two decades, we and others have characterized the structure and/or functional properties of ten of these orphan clades (Table 1). We also added several new members to the previously identified clades. Since then, sequences of a large number of 3FTxs have been determined by high-throughput transcriptomics and proteomics. Currently, more than 1700 amino acid sequences of snake venom 3FTxs are deposited in various free-access databases (e.g., UniProt and NCBI) and new members are constantly being added. Based on the sequence analyses, we classified all potential neurotoxins (for details, see ref. [36]). However, no unified update has integrated the post-omics explosion of sequences with a systematic reclassification of the remaining uncharacterized 3FTxs. By preliminary sequence analyses, we identified over 550 3FTxs whose functions have not yet been determined. In this review, we briefly summarize the data obtained for orphan 3FTxs during the last two decades. We have classified the newly discovered 3FTxs and updated the groups of orphan toxins to 157 different clades based on their sequence similarity. We also codify structural features of 3FTxs such as N-terminal and C-terminal length, loop sizes, and interloop connectivity into coherent architectural categories. We anticipate that this updated and consolidated “orphan 3FTx toolkit” will be useful in providing a clearer picture of the unexplored library of novel biological ligands with potential new structures and functions.

## 2. De-Orphaned 3FTxs

**Figure 2:**
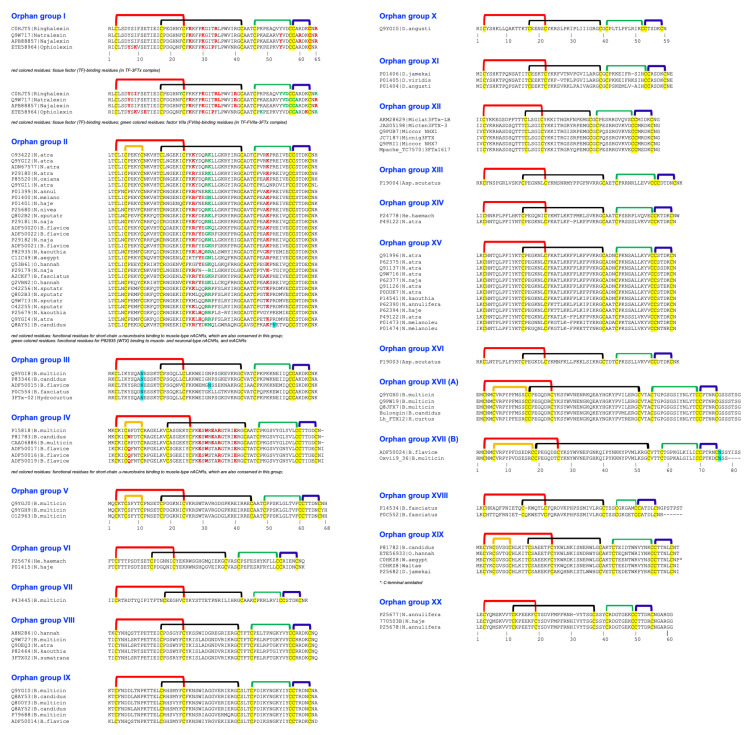
Multiple sequence alignment of the 20 groups of orphan 3FTxs as classified in 2003 [2] with recently reported sequences added as new members. NCBI/UniProt accession numbers are indicated. Cys residues are highlighted in yellow with disulfide bridges indicated above. The first disulfide bridge (S-S) is colored red, the second S-S is colored black, the third S-S is colored green, the fourth S-S is colored blue, the fifth S-S is colored orange. The same color code for S-S is consistently used throughout the manuscript. Predicted N-linked glycosylation sites (Ans-Xaa-Ser/Thr) are highlighted in cyan. Additional color codes are explained in the note immediately under the relevant sequences.

In 2003, we described twenty clades of orphan toxins by sequence analyses (Figure 2, Table 1) [2]. In the last two decades, the structure and/or function of some toxins from various orphan clades have been characterized. We will describe details of these findings to inspire further research in other clades.

### 2.1. Orphan Group I: Righalexin-Related Toxins (Extrinsic Tenase Inhibitors—Exins)

When first described in 2003, the orphan group I 3FTxs had a single member identified as neurotoxin-like protein from *Naja atra* venom (NTL2) (NCBI #Q9W717) [2]. In 2016, we identified and characterized ringhalexin (NCBI #C0HJT5), a novel toxin from *Hemachatus haemachatus* venom [37], that shared 94% identity (98% similarity) with this uncharacterized NTL2. Ringhalexin showed potent anticoagulant activity by inhibiting the activation of factor X by the tissue factor (TF)–activated factor VII (FVIIa) complex. We also determined its three-dimensional structure (PDB: 4ZQY); this is the first structure of a 3FTx with anticoagulant properties [37]. We identified two other members of this group—one transcript from a *Naja naja* venom gland (najalexin, NCBI #APB88857) [20,38] and the other from *Ophiophagus hannah* (ophiolexin, NCBI #ETE58964) [39]. They shared 94% and 84% sequence identity with ringhalexin. We renamed NTL2 from *Naja atra* as natralexin. Natralexin is identical to najalexin (Figure 2). We evaluated the interaction of this group of 3FTxs with all components of the TF-FVIIa complex using in silico protein–protein docking studies [20]. These docking studies revealed amino acid residues crucial for their interaction with TF and TF-FVIIa (for details, see ref. [20]) (Figure 1c). Furthermore, these functional residues are not found in other groups of 3FTxs, which exhibit distinct pharmacological properties. Thus, we delineated the distinct functional sites of these toxins using docking studies on the TF and TF-FVIIa complex (Figure 2), and the target and function of orphan group I 3FTxs, consisting of four members (Table 1), have been described. Interestingly, ringhalexin also exhibited weak, irreversible neurotoxicity by interacting with the nicotinic acetylcholine receptor (nAChR). Further studies are needed to define nAChR subtype selectivity and the amino acid residues involved in their interaction with nAChR(s).

### 2.2. Orphan Group II (Antagonists of nAChRs and mAChRs)

This was the largest group of orphan toxins with 17 members [2], and we have added 12 new members (Table 1). This group contained toxins that were classified as “weak neurotoxins” [40,41] as they exhibit lower toxicity (median lethal dose (LD_50_), 5–80 mg/kg) in contrast to prototype α-neurotoxins (LD_50_, 0.04–0.3 mg/kg) [42,43,44,45]. These toxins contain five disulfide bridges. In addition to the four conserved disulfide bridges, the fifth disulfide bridge is located at the tip of loop I, unlike α- and κ-neurotoxins which have the fifth disulfide bridge in loop II. The weak toxin from *Naja kaouthia* venom blocks the binding of α-bungarotoxin to *Torpedo* muscle-type nAChR (half-maximal inhibitory concentration (IC_50_), 2.2 μM) and inhibits acetylcholine-induced current of rat and human α7 nAChRs (IC_50_s, 8.3 μM and 15 μM). Previously, we classified this group as group II orphan toxins, as its specific target was not known [41]. Subsequent studies have shown that this toxin also interacts with muscarinic acetylcholine rectors (mAChRs) at micromolar concentrations [46,47]. The three-dimensional structures of some of these toxins have been determined (Protein Data Bank (PDB) entries: 2MJO, 2JQP). The amino acid residues involved in the interaction with nAChRs and mAChRs have been identified [47,48]. Thus, significant progress has been made in understanding the structure and function of this group of toxins.

### 2.3. Orphan Group III: Bucain-Related Toxins

There were two members in this group [2], and we have added two additional members (Table 1). The three-dimensional structures of bucain have been determined by both X-ray crystallography (PDB: 2H8U, ref. [49]) and nuclear magnetic resonance (NMR) spectroscopy techniques (PDB: 1VYC). Although Asn11 is a potential *N*-glycosylation site, native bucain is not glycosylated. As of now, the functional characteristics of these toxins remain unknown.

### 2.4. Orphan Group IV: Candoxin-Related Toxins (Antagonists of nAChRs)

In 2003, we identified two 3FTxs in this group [2]. We characterized P81783 from *Bungarus candidus* venom and it was named candoxin [34,50,51]. Structurally, candoxin has 66 amino acid residues, including 10 cysteine residues that form five disulfide bonds. In addition to the four conserved disulfide bonds in the central core, the fifth disulfide bridge in candoxin is located at the tip of loop I, instead of loop II as found in α-neurotoxins and κ-neurotoxins. It targets nAChRs and shows potent postsynaptic neurotoxicity in chick biventer cervicis muscle (CBCM) preparations [50]. It strongly inhibits acetylcholine (ACh)-evoked currents in the rat muscle-type (α1βγδ) receptors with an IC_50_ of 10 nM. This inhibition is rapidly and fully reversible with a 10 min wash. In contrast, its inhibition of rat α7 nAChR (IC_50_, 50 nM) is irreversible [50]. We also determined its three-dimensional structure using NMR methods (PDB: 1JGK, ref. [51]). Candoxin shows a remarkable similarity in the spatial (topological) disposition of the critical, functional residues for the muscle receptor to erabutoxin-a and α-cobratoxin [50]. Thus, potent neurotoxicity is most likely due to the presence of pharmacophores similar to those found in α-neurotoxins. We have added four new members to this group (Table 1).

Candoxin specifically targets glial cells in the central nervous system due to its high affinity and irreversible binding to α7 nAChR [52]. Using the functional site of candoxin (17-residue loop II peptide, CDX), a drug delivery system that traverses the blood–brain barrier (BBB) was developed [53,54,55]. CDX-coated micelles/liposomes deliver the drug paclitaxel to glioblastoma multiforme (GBM) tumors in mice. The same group developed red blood cell membrane-coated nanoparticles with CDX, which retain the biological functions of natural cell membranes and allow prolonged blood circulation with low immunogenicity [56]. The doxorubicin-loaded nanoparticles show superior therapeutic efficacy with reduced toxicity compared to the non-targeted drug formulations. Thus, candoxin has provided an opportunity in a highly selective drug delivery system.

We classified the group of 3FTxs with the fifth disulfide in loop I as non-conventional toxins [57]. The weak neurotoxins (orphan group II described above) among non-conventional toxins have further substitutions of functional residues responsible for their interactions with nAChRs leading to the reduced neurotoxic potency.

### 2.5. Orphan Group V: γ-Bungaratoxin-Related Toxins

There were three members in this group [2]. Interestingly, isolated from *Bungarus multicinctus* venom, a member of this group shows potent lethality (LD_50_, 0.15 mg/kg) [58], which is comparable to that of α-neurotoxins. This toxin has the RGD tripeptide sequence in loop II that plays an important role in adhesion. Despite this RGD sequence, γ-bungarotoxin inhibits platelet aggregation with an IC_50_ of 34 μM [17]. Based on the NMR structure (PDB: 1MR6), the authors showed that the loop size may be responsible for the three orders of magnitude lower antiplatelet activity (Figure 1b). Interestingly, γ-bungarotoxin impairs the vascular endothelial barrier function by inhibiting integrin α5 and promoting the permeability [59]. However, the mechanism of potent toxicity of γ-bungarotoxin is not yet known.

### 2.6. Orphan Group VIII: Haditoxin-Related Toxins (Dimeric Antagonists of nAChRs)

There were three members in this group [2], and we have added one new member (Table 1). We characterized A8N286 from *Ophiophagus hannah* venom and named it haditoxin [32]. It is a postsynaptic neurotoxin that belongs to short-chain 3FTx family and retains functionally important Gln7, Ser8, Trp29, Asp31, Arg33, Arg36, and Glu38 residues that are found in α-neurotoxins [60,61]. Unlike monomeric short-chain 3FTxs, haditoxin is a non-covalent homodimer. It forms an antiparallel dimer similar to κ-neurotoxins (long-chain neurotoxins) [62]. However, the natures of interactions and the residues involved in dimerization in haditoxin and κ-neurotoxins are distinct (PDB: 3HH7) [32]. Haditoxin binds to muscle-type nAChR (unlike κ-neurotoxins) as well as neuronal (α7, α3β2, and α4β2) nAChRs (similar to long-chain neurotoxins). The other members in this group retain the most amino acid residues involved in dimerization, but the functional site residues are substituted by non-conserved residues (Figure 2). It will be interesting to understand the subtype selectivity and structure–function relationships.

### 2.7. Orphan Group XV: Cytotoxin-A5- and μ-EPTX-Na1a-Related Toxins (Integrin/Sodium Channel Inhibitors)

There were ten members in this group [2], and we have added three new members (Table 1). In 2006, Wu et al. showed that cytotoxin A5 (CTX A5; Q91996/P62375), a member of this group, binds to αvβ3 integrin and inhibits bone resorption [63]. αvβ3 integrin mediates the formation of multinucleate osteoclasts and plays a key role in the late stage of their differentiation and adhesion process during bone resorption [64,65]. Antagonists of αvβ3 based on the RGD sequence inhibit bone resorption in vitro and prevent osteoporosis in vivo [66,67]. Among six CTXs, CTX A5 bound to αvβ3 with an apparent dissociation constant of ~0.3 μM [63]. Mn^2+^ (10 mM) or Mg^2+^ (10 mM) enhanced the binding of αvβ3 to CTX A5 by 5- and 2-fold, respectively. CTX A5 inhibits murine osteoclast differentiation by perturbing polykaryon fusion and bone resorption through binding to αvβ3. It is interesting to note that, despite the absence of the RGD sequence, CTX A5 binds to αvβ3 integrin.

The newly added P0DUK7 toxin from *Naja atra* venom (named as μ-EPTX Na1a or simply Na1a) is a potent inhibitor of voltage-gated sodium channel (VGSC or Na_v_) 1.8 [68], a key target for pain control [69,70,71]. Na1a potently inhibited tetrodotoxin (TTX)-resistant Na^+^ current in rat dorsal root ganglion (DRG) neurons with an IC_50_ of 141 nM. The toxin is a gating modifier targeting Na_v_1.8. It inhibits human Na_v_1.8 expressed in cell lines (HEK-293 cells: IC_50_, 380 nM), Na_v_1.5 (ND7/23 cells: IC_50_, 8.5 μM), and Na_v_1.4 (ND7/23 cells: IC_50_, >10 μM) [68]. Human Na_v_1.2, Na_v_1.3, Na_v_1.6, and Na_v_1.7 currents are unaffected even at a 10 mM concentration. Thus, Na1a is a potent and selective inhibitor of Na_v_1.8. It shows no effect on other pain targets, such as transient receptor potential channel (TRP) subtypes TRPV1, TRPV2, TRPV3, TRPV4, TRPA1, TRPM8, and TRPC3–6 at 10 μM. Na1a shows significantly better analgesic effects than morphine when the doses were 7 and 70 nmol/kg in acute pain models of writhing and biphasic inflammation mouse models. It also shows potent analgesic effects compared to morphine in chronic pain models (complete-Freund’s-adjuvant-induced inflammatory and neuropathic chronic pain and partial-nerve-ligation-induced neuropathic chronic pain) [68]. Thus, Na1a is an excellent lead for developing pain therapeutics. Interestingly, there is only a single residue difference between CTX A5 and Na1a; Val36 is replaced by Phe36 in Na1a (Figure 2). CTX A5 binds to αvβ3 (see above), and αvβ3 integrin signaling is essential for proliferation and survival of endothelial cells, such as human umbilical vein endothelial cells (HUVECs) and calf pulmonary artery endothelial (C-PAE) cells. Inhibition of αvβ3 integrin leads to HUVEC and C-PAE cell apoptosis, whereas its activation promotes HUVEC proliferation [72,73,74]. Na1a does not promote or inhibit proliferation of HUVECs and C-PAE cells [68]. Therefore, Na1a may not bind to αvβ3 integrin. Thus, it is intriguing to understand molecular details of interactions of CTX A5 and Na1a with αvβ3 integrin and Na_v_1.8 and other VGSCs. The other members in this group show subtle differences compared to CTX A5 and Na1a and, hence, may have different selectivity profiles against various integrins and VGSCs.

### 2.8. Orphan Group XVII: BM8/BM14/Bulongin-Related Toxins (Antagonists of mAChRs and nAChRs)

There were three members in this group [2], and we have added one new member (Table 1). All the members were found in the venoms of the genus *Bungarus*. The first member was identified by complementary DNA (cDNA) sequencing of *B*. *multicinctus* venom glands [75]. Two native proteins (BM8 and BM14) were purified from the same venom. BM8 showed a protein sequence identical to that deduced from the cDNA sequence [75], while Glu37-Ala38 in BM8 (Q9PW19) is replaced by Lys37-Lys38 in BM14 (Q8JFX7). We purified a similar toxin from *Bungarus candidus* (Indonesia) venom and sequenced by Edman degradation. This toxin shows substitutions at three positions (Lys38Glu, Ile46Met, and Thr56Ser) compared to BM14 (R. M. Kini, unpublished observations). This toxin was named bulongin (*Bungarus* long toxin) because of its unusually long loops. BM14, but not BM8, inhibits the binding of [^3^H]quinuclidinyl benzilate to the M2 but not M1 type of mAChR [76]. Modification of Lys37 and Lys38 of BM14 abolished its M2 binding, suggesting a role for these Lys residues. Interestingly, BM8 and BM14 do not inhibit α-bungarotoxin binding to *Torpedo californica* nAChR at concentrations up to 100 μM [76]. In contrast, Utkin et al. showed that BM8 inhibits α-bungarotoxin binding to *T. californica* nAChR (IC_50_, 31 nM) and to human α7 nAChR (IC_50_, 43 nM) [77].

We classified these toxins into two subgroups. Group XVII (A) contains all three toxins described above, whereas the two new toxins in XVII (B) have subtle sequence differences in all three loops compared to XVII (A) toxins and are expected to differ significantly in their functions.

### 2.9. Orphan Group XIX: Bucandin/Actiflagelin-Related Toxins

There were two members in this group [2], and we have added two new members (Table 1). We determined the three-dimensional structure of bucandin isolated from *B. candidus* venom using X-ray crystallography and NMR methods (PDB: 1F94 and 1IJC) [78,79]. Actiflagelin was isolated from *Walterinnesia aegyptia* venom [25]. It shows 85.7% identity and 90.5% similarity with bucandin. Interestingly, the C-terminal of actiflagelin is amidated (-CONH_2_) [25], whereas that of bucandin is a free carboxylic acid (-COOH) [78,79]. Actiflagelin activates the motility of sperms from OF1 male mice by 27.3% in vitro [25]. The target receptor of actiflagelin and its structure–function relationships have yet to be determined.

### 2.10. Orphan Group XX

There were three members in this group [2], and we have added a new member (Table 1). The toxin P01402 from *Hemachatus haemachatus* venom shows similarity to exactin [80], which was isolated from same venom and belongs to Ω-neurotoxins. This toxin has been removed from this group. Nakoroxin [81] from *Naja kaouthia* venom is also similar to exactin and other Ω-neurotoxins (described below). Currently, details of the structure or function of toxins of this group of toxins are not yet characterized.

### 2.11. Other Orphan Groups

Significant progress has been made in the last two decades on various orphan toxin groups that were identified [2]. The results suggest that these efforts have led to discovery of some novel target receptors, ion channels, or enzymes and functions, leading to potential therapeutic applications and drug delivery. Not many efforts have been made towards resolving the structure and function of toxins from several groups. We have added new toxin members to all of these groups (Table 1).

## 3. Novel Classes of 3FTxs

Several 3FTxs with interesting biological activities and structures were isolated and characterized in the last two decades. Here are most of these novel 3FTxs.

### 3.1. Colubrid 3FTxs

We isolated the first postsynaptic neurotoxin (α-colubritoxin) from a “non-venomous” colubrid, *Coelognathus radiatus* (formerly *Elaphae radiata*) [82]. We compared toxin profiles of *C. radiatus* (Colubrinae), *Cerberus rhynchops* (Homalopsinae), *Heterodon nasicus* (Xenodontinae), *Leioheterodon madagascariensis* (Pseudoxyrhophiinae), and *Psammophis mossambica* (Psammophiinae) with those of *Ophiophagus hannah* (Elapidae) and *Atheris squamiger* (Viperidae) venoms and identified the toxin-like molecular masses. The major toxin with 8498 Da mass was purified from *C. radiatus* venom. Unlike elapid and hydrophiid 3FTxs, the N-terminus of this toxin was blocked. After pyroglutamase treatment, we were able to sequence the N-terminal region of the protein by Edman degradation [82]. The complete sequence of the protein was determined by protein sequencing. α-Colubritoxin has a long N-terminal segment with an additional seven residues compared to typical 3FTxs. It has 10 Cys residues forming five disulfide bridges (four conserved and the fifth disulfide bridge located in loop I). It showed reversible, postsynaptic neurotoxicity in CBCM. This was the first evidence for the presence of a 3FTx with neurotoxicity in “non-venomous” snakes and supports the true venomous function as part of the ancestral biological role of Duvernoy’s gland [82]. This paper, along with the molecular phylogenetics paper [2], were adjudged as the joint best papers for 2003 in the *Journal of Molecular Evolution*, and Bryan Fry received the Zuckerkandl prize.

We evaluated toxin profiles of 42 snake venoms: fifteen species each from Colubrinae and Elapidae, four species from Viperidae, two species each from Homalopsinae and Xenodontinae, and one species each from Atractaspididae, Natricinae, Psammophiinae, and Pseudoxyrhophiinae using mass spectrometry [83]. Most of these venoms are previously unstudied or understudied types. Thus, this study provided a comprehensive overview of the presence or absence of various toxin classes and surprising details of toxin types in colubrid venoms and described the clinical and evolutionary impact of the toxin profiles [83]. Further, we studied the in vitro neuromuscular activity of “colubrid” venoms from Colubrinae, Homalopsinae, Natricinae, Pseudoxyrhophiinae, and Psammophiinae families in the CBCM preparations. *Boiga dendrophila*, *B. cynodon*, *B. dendrophila*, *B. dendrophila gemincincta*, *B. drapiezii*, *B. irregularis*, *B. nigriceps,* and *Telescopus dhara* venoms display irreversible postsynaptic neuromuscular activity, whereas *Ahaetulla prasina*, *Enhydris chinensis*, and *Leioheterodon madagascariensis* venoms exhibit no significant neurotoxicity [84]. Interestingly, neostigmine reverses the inhibition by *B. cynodon* venom, while the effect of *Psammophis mossambicus* venom reverses spontaneously. Thus, these venoms contain neurotoxins with distinct reversible modes of neurotoxicity, and envenomation from some of these species may have significant clinical and evolutionary implications [84]. *B. dendrophila* venom lacks activity in the epididymal segment of rat vas deferens smooth muscle preparation, while it inhibits electrically evoked twitches in the prostatic segment [85]. This inhibition may be due to adenosine α1 receptor activity, most likely mediated by adenosine. In guinea pig ileum, the venom induces concentration-dependent contractions that are mediated by the muscarinic receptor [85]. *B. dendrophila* venom induces a significant decrease in the mean arterial pressure without affecting the heart rate. Thus, *B. dendrophila* venom has components that affect nicotinic, purinergic, and muscarinic receptors [85].

#### 3.1.1. Denmotoxin

We purified and characterized a novel toxin (denmotoxin) from the venom of *B. dendrophila* (mangrove catsnake) which preys primarily on birds [86]. The toxin sequence was completed using protein and cDNA sequencing methods. This 77-amino-acid-residue toxin has an extended N-terminus and five disulfide bridges, similar to α-colubritoxin. However, it shows only ~50% identity with α-colubritoxin and <30% identity with elapid 3FTxs. Chemically synthesized and folded denmotoxin shows similar elution profile, mass spectrum, circular dichroism, and pharmacological functions to the native denmotoxin [86]. This *B. dendrophila* monomeric toxin was named denmotoxin. It shows potent, irreversible postsynaptic neurotoxicity in CBCM, ~10-fold less potent than α-bungarotoxin. In contrast, denmotoxin shows reversible, 100-fold less potent neurotoxicity in mouse hemidiaphragm (MHD) preparation, while α-bungarotoxin shows irreversible, equally potent neurotoxicity in MHD compared to CBCM. Denmotoxin does not cause paralysis or other biological effects in mice after intraperitoneal or intracerebroventricular injection of the toxin at up to 20 and 10 mg/kg, respectively. Thus, denmotoxin shows remarkable species specificity with a preference for avian neuromuscular junctions [86]. We determined the three-dimensional structure of denmotoxin by X-ray crystallography (PDB: 2H5F). Overall, the denmotoxin structure is similar to those of other 3FTxs. This non-conventional toxin displays unique features, such as the twisted tip of the central loop, which originates from a Pro40-induced kink. The unusually long N-terminal segment is unstructured and probably flips above the core of the molecule. The tip of the central loop is electronegative, unlike those of other neurotoxins in the 3FTx family. This is the first high-resolution structure of a colubrid 3FTx [86]. The functional site residues involved in the interaction with nAChRs in erabutoxin and cobratoxin are all replaced in denmotoxin. Even the all-important positively charged Arg33 is replaced by the negatively charged Asp41. Despite such severe changes in the structure, denmotoxin retains high affinity binding to chicken nAChRs. To understand avian muscle receptor selectivity, we focused on substitutions in the loop regions in close proximity to the toxin binding site in the mouse and chick muscle (α1βγδ) nAChRs. Functional loops A and B from α1 and loop F from γ- and δ-subunits are identical in both species. However, loop C from α1 and loops D and E from the γ- and δ-subunits show several changes, which may contribute to species-dependent receptor susceptibility. The complex structure of α-cobratoxin with acetylcholine binding protein (AChBP) from *Lymnaea stagnalis*, a surrogate for the ligand binding extracellular domain (ECD) of nAChRs, reveals toxin–receptor interactions [87]. The unstructured region between β8 and β9 is involved in the interaction. This segment is equivalent to the complementary face of the γ- and δ-subunits of muscle nAChRs. Interestingly, only the chick δ-subunit, but not the mouse δ-subunit, has Arg193 just in front of loop F. We proposed that this Arg193 may contribute to electrostatic interactions with denmotoxin Asp41, causing enhanced and irreversible binding of the toxin to the chick muscle nAChR [86]. This indirectly suggests that denmotoxin binds to the δ-subunit of the muscle nAChR, unlike canonical α-neurotoxins, which bind to the α1-subunit [60,61]. We determined the complete mRNA and gene sequences of denmotoxin [88]. The denmotoxin gene has four exons compared to the three exons in other 3FTx genes. The new second exon encodes most of the 15-mer propeptide and the N-terminal extension [88]. The propeptide region is removed by a proteolytic cleavage processing on the N-terminal side of Gln. The newly formed N-terminal Gln is cyclized by glutaminyl cyclase [89]. This pyroglutamic acid protects the long, flailing N-terminal end from proteolytic degradation by aminopeptidases.

Since then, several monomeric colubrid neurotoxin sequences have been found. Interestingly, these toxins have variable propeptide sequences and proteolytic processing sites. Recently, we classified 162 colubrid monomeric toxins into seven classes. Class 1 toxins were further subdivided (for details, see ref. [36]). Additionally, a few toxins, such as sulmotoxin and fulmotoxin, have been characterized, and they also show species selectivity [90,91]. In some of these toxins, either Pro40 (inducing the kink in the central loop) or Asp41 (probably involved in species selectivity) is replaced. It is intriguing to understand the impact of substituting Asp41 with Asn41 (acidic to neutral) or Asp41 with His41 (charge reversal from negative to positive or introduction of aromaticity) or deletion of a few residues from the central loop (at times including both Pro40 and Asp41) on species selectivity and neurotoxic function. Detailed studies of the toxins, their pharmacological properties, and gene sequences will provide greater understanding of these unique molecules.

#### 3.1.2. Irditoxin

A novel heterodimeric three-finger neurotoxin was isolated from the venom of the brown tree snake *Boiga irregularis*. This *B. irregularis* dimeric toxin was named irditoxin [31]. The subunits are covalently linked by a single disulfide bridge. The sequences of these subunits were determined by Edman degradation and cDNA sequencing. The deduced amino acid sequence has an additional 34 residues, composed of a 19-residue signal peptide and a 15-residue propeptide, at the N-terminus compared to the mature protein. Similar to denmotoxin, both subunits have a seven-residue extension compared to typical 3FTxs. Both subunits have 11 Cys residues; 10 Cys residues form five disulfide bridges, similar to non-conventional 3FTxs. The 11th, or spare, Cys residue is located at the tip of loop II in subunit A and loop I in subunit B. This is the first covalently linked heterodimeric 3FTx described [31]. We determined the three-dimensional structure of irditoxin by X-ray crystallography (PDB: 2H7Z). Both subunits, similar to denmotoxin [86], retain all key structurally invariant residues found in 3FTxs [92,93] and fold similarly to elapid 3FTxs. As with denmotoxin, both subunits have a twisted tip at the central loop, which originates from the kink induced by Pro38A and Pro40B. The long N-termini are unstructured and are located above the core of the molecule. The two monomers are placed in a unique diagonal geometry, resulting in a flattened topology with two distinct molecular faces. Loop IIIA, closely associated with loop IB, is inaccessible for receptor interactions, while loops IA, IIB, and IIIB are accessible. The dimeric interface is distinctly different compared to the dimeric interfaces of κ-bungarotoxin, haditoxin, and fulditoxin [29,30,62]. Irditoxin induces rapid flaccid paralysis, dyspnea, and increased respiratory rate in *Hemidactylus* geckos from 0.1 to 10 mg/g. The geckos die within 10 min to 3 h at doses above 1.0 mg/g (LD_50_, 0.55 mg/g). At sublethal doses (0.3 mg/g and below), they were immobilized for up to 3 days before full recovery. Irditoxin induces a rapid onset of inactivity, dyspnea, neck droop, and death (LD_50_, 0.22 mg/g) in chicks [31]. In contrast, irditoxin does not induce any effects in mice at doses up to 25 mg/g (intraperitoneal (i.p.)). Thus, irditoxin shows typical peripheral neurotoxicity in a species-specific manner. Irditoxin shows potent, irreversible, postsynaptic neurotoxicity in CBCM preparation with an IC_50_ of 11.2 nM, almost identical to α-bungarotoxin (IC_50_, 11.4 nM). In contrast, irditoxin is about three orders of magnitude less active, but still irreversible, in rat hemidiaphragm (RHD) preparation [31]. α-Bungarotoxin shows irreversible toxicity with an IC_50_ of 96 nM in RHD preparation. In competitive experiments with radiolabeled ligands, irditoxin shows weak binding to mouse muscle α1βγδ nAChRs (at μM concentrations) but not to neuronal α7 and α3β2 nAChRs. Although both subunits lack functional residues of α-neurotoxins [60,61], they have equivalents of the Pro40-Asn41 residues in denmotoxin, which were predicted to bind to Arg193 of the δ-subunit of chick muscle nAChRs [86]. As only the tip of loop IIB is free for interaction with the target receptor, we propose that Asp41B probably drives the irreversible binding to chick nAChRs. The flat dimeric surface may provide an extended binding surface making irditoxin an irreversible antagonist in both avian and mammalian muscle nAChRs.

Sulditoxin, another dimeric neurotoxin, was isolated from *Spilotes sulphureus* venom [91]. Several sequences of potential A and B subunits have been found in the transcriptomes of colubrid Duvernoy’s glands. There are 47 and 36 subunits A and B transcripts, respectively, compared to 162 transcripts for monomeric toxins. They have variable propeptide sequences and proteolytic processing sites, but not as many distinct ones as are found in monomeric toxins. Recently, we classified them into three and five groups, respectively (for details, see ref. [36]). It is fascinating to consider the impact of substitution or deletion of Pro40 and/or Asn41 on species selectivity and neurotoxic function.

The sequences of three toxins contain spare Cys residues in both loops I and II, similar to the subunits of heterodimeric toxins. For interaction with nAChRs, the tip of the central loop is essential. Therefore, we do not expect the spare Cys residue to form heterodimers as the interchain disulfide bridges will sterically interfere with receptor interaction. Instead, we hypothesize that these two Cys residues form intrachain disulfide bridges.

### 3.2. Adrenoceptor Inhibitors

G-protein coupled receptors (GPCRs) are the largest family of membrane proteins and they serve as targets for about 50% of currently used drugs. Adrenergic receptors (or adrenoceptors), a class of GPCRs, are divided into three classes (α1, α2, and β) and α1-adrenoceptors into three subtypes (α_1A_, α_1B_, and α_1D_) [94]. Here, we will describe the discovery, characterization, and functions of adrenoceptor modulators from the 3FTx family.

#### 3.2.1. β-Cardiotoxin

We created a partial cDNA library with a focus on low-molecular-weight components of the *Ophiophagus hannah* venom gland by picking 346 clones with 100–800 bp [95]. In addition to the most abundant clones that encoded long neurotoxin 1, we identified five new 3FTx clones. The protein encoded by one of these clones (AY354198) shows high sequence identity with five proposed cardiotoxin precursors. However, they showed only 55–65% identity with canonical CTXs and CTX-like basic proteins (with most differences in loops) and even lower identity with neurotoxins. We isolated and purified the protein from the crude venom, targeting the 7012.42 Da component [95]. The protein was not lethal to mice at 10 mg/kg (i.p.). At 100 mg/kg, the mice show labored breathing, impaired locomotion, lack of response to external stimuli, and finally die after ~30 min. There was no internal hemorrhage or visible damage to internal organs. The protein does not show anticoagulant or hemolytic activity. The electrocardiogram (ECG) data show that the protein, administered by tail vein, decreases heart rate in anesthetized rats [95]. This decrease in heart rate is dose-dependent. In contrast, canonical cardiotoxin CM18 from *Naja atra* venom increases heart rate. Thus, CM18 induces a positive chronotropic effect (tachycardia), while our protein shows a negative chronotropic effect (bradycardia). The protein induces negative chronotropic effects in Langendorff preparations of isolated perfused hearts without significant change in contractility (inotropism), as indicated by the left ventricle developed pressure. Thus, the protein likely induces its effects through direct action on cardiac muscles [95]. The protein competitively displaces radioligand bound to human β1- and β2-adrenoceptors, with IC_50_s of 5.3 μM and 2.3 μM, respectively. As β-adrenoceptors and the adrenergic signaling cascade are responsible for the control of heart rate [96], we proposed that this protein causes a negative chronotropic effect on heart rate by binding to β1-adrenoceptors in cardiomyocytes [95]. The observed respiratory symptoms in mice are due to its interaction with β2-adrenoceptors in the bronchi, resulting in bronco-constriction. Because of its interaction with β-adrenoceptors, the protein was named β-cardiotoxin. To our knowledge, this is the first exogenous protein beta-blocker isolated from nature [95]. All currently used beta-blockers are (aryloxy)propanolamines. Beta-blockers are the drugs of choice in the treatment of cardiovascular diseases, particularly high blood pressure and myocardial infarction [97]. They are also used in the treatment of ventricular arrhythmias, heart failure, digitalis intoxication, and fetal tachycardia. Since β-adrenoceptors are also found in various non-cardiac tissues, beta-blockers are used to treat migraines, essential tremor, situational anxiety, alcohol withdrawal, hyperparathyroidism, glaucoma, portal hypertension, and gastrointestinal bleeding [98].

#### 3.2.2. AdTx1 (ρ-Da1a)

With a targeted search for fraction(s) from *Dendroaspis angusticeps* crude venom to inhibit ^3^H-prazosin binding (α_1A_-adrenoceptor) to rat brain synaptosomes (RBSs), Quinton et al. isolated and purified a toxin (P85092) [99]. Its amino acid sequence was ascertained by MS/MS of seven tryptic peptides. The toxin, named AdTx1, was chemically synthesized, folded, and used for further studies. They used prazosin for α1-adrenoceptors, rauwolscine for α2-adrenoceptors, CGP-12177 for β-adrenoceptors, and N-methyl-scopolamine (NMS) for muscarinic receptors as selective orthosteric ligands. AdTx1 inhibits ^3^H-prazosin binding with two binding sites: high-affinity (IC_50_ 4 nM) and low-affinity (IC_50_ 1500 nM) sites [99]. It inhibits rauwolscine binding in micromolar concentrations and fails to inhibit CGP-12177 and NMS binding at 10 μM AdTx1. AdTx1 inhibits ^3^H-prazosin binding to human α_1A_-adrenoceptor on yeast membranes, with an IC_50_ value of 1.1 nM (inhibitory constant (Ki) = 0.35 nM). In contrast, AdTx1 shows low affinity for human α_1B_-adrenoceptor (IC_50_, 950 nM) and rat α_1D_-adrenoceptor (IC_50_, 1250 nM) subtypes [99]. ^125^I-AdTx1 shows specific binding to human α_1A_-adrenoceptors on yeast membranes but not to human α_1B_- and rat α_1D_-adrenoceptors. AdTx1 shows slow association (K_on_, 6.4 × 10^6^·M^−1^·min^−1^) and dissociation (K_off_, 0.192 h^−1^) kinetics. In rabbit isolated prostate gland smooth muscle, agonist-induced contraction is mediated mainly by activation of α_1A_-adrenoceptor [100]. AdTx1 decreases both potency and efficacy of phenylephrine in a concentration-dependent manner. AdTx1 reduces maximal contractions to 58.8% (30 nM) and 14.0% (100 nM) induced by phenylephrine and thus displays insurmountable antagonism. The high affinity and selectivity to α_1A_-adrenoceptor, together with the peptide-based structure and long half-life of the AdTx1/α_1A_-adrenoceptor complex, offer opportunities for the development of improved pharmacological tools. The long half-life of the AdTx1-α_1A_-adrenoceptor complex could lead to a dose-dependent, long-lasting blockade of α_1A_-adrenoceptor, reducing receptor density and consequently decreasing pEC_50_ and agonist efficacy. α_1A_-Adrenoceptor antagonists are considered the most effective monotherapy for benign prostatic hyperplasia (BPH).

ρ-Da1a (AdTx1) at 1 μM does not show significant agonist or antagonist activity on 78 GPCRs and eight principal ion channels implicated in cardiac activity [101]. It is a non-competitive antagonist of α_1A_-adrenoceptor (Figure 1g). In human isolated prostate gland smooth muscle, ρ-Da1a (100 nM) decreases both potency and efficacy of noradrenaline. ρ-Da1a reduces maximal contractions to 26.1% (100 nM) and 9.2% (300 nM) induced by noradrenaline and thus displays insurmountable antagonism. Tamsulosin, one of the most potent α_1A_-adrenoceptor antagonists, at 10 nM almost fully reduces maximal contractions [102]. Tamsulosin, unlike ρ-Da1a, fails to completely inhibit the agonist response [101,103]. Thus, the nature of the antagonism by ρ-Da1a and tamsulosin differs. ρ-Da1a dose-dependently abrogates the effects of phenylephrine on intraurethral pressure (IUP). On a molar basis, tamsulosin is three times more potent than ρ-Da1a when administered intravenously (i.v.) in anesthetized rats. The longer duration of action of ρ-Da1a may overcome this difference [101]. Unlike tamsulosin, orally administered ρ-Da1a fails to show any effects. In addition to the classical use of α_1A_-adrenoceptor antagonists for the treatment of BPH, ρ-Da1a may be useful in treating painful bladder syndrome and in facilitating the passage of renal stones from the ureter to the urinary bladder.

#### 3.2.3. ρ-Da1b

Rouget et al. used ^3^H-rauwolscine competition binding assays for α2-adrenoceptors to isolate and purify ρ-Da1b from *Dendroaspis angusticeps* venom [104]. Its amino acid sequence was determined by a combination of Edman degradation and mass spectrometry (P86419). It interacts with human α2-adrenoceptors with an affinity of 14 nM for the α_2A_-adrenoceptor and with a weak selectivity for α_2B_-adrenoceptor (73 nM) and α_2C_-adrenoceptor (38 nM). It shows poor binding to human α_1A_-adrenoceptor (2.1 μM) and fails to bind to other α_1_- and β-subtypes. The toxin does not fully displace rauwolscine, leaving the same level of residual binding in all three α2-subtypes. The α_1A_-adrenoceptor is implicated in Parkinson’s disease [105], epilepsy, and depression [106], as well as in diabetes [107], intestinal motility [108], orthostatic hypotension [109], cardiac function [110], and pain [111]. ρ-Da1b, as an antagonist, may become a useful tool in the study of α2-adrenoceptor physiology and in the development of novel drug candidates.

#### 3.2.4. α-Adrenergic/Muscarinic/Dopaminergic/Aminergic Toxins

Interestingly, the amino acid sequences of α-adrenoceptor inhibitors show strong identity and similarity to those of muscarinic toxins. Koivula et al. showed that the muscarinic toxin MTα selectively antagonizes the α_2B_-adrenoceptor [112]. Subsequently, adrenoceptor activity of several other muscarinic toxins was characterized [7,113,114]. Fruchart-Gaillard et al. using a rational “loop grafting” design method, engineered several chimeras, and evaluated their gain of function towards the M1 and M4 muscarinic receptors and the α_1A_-adrenoceptor [115]. With the crystal structures of MT1 and two chimeras, the authors defined the molecular details underlying the functional gains. Thus, their efforts suggest the possibility of designing 3FTxs with novel pharmacological profiles through loop permutation [115].

### 3.3. Viperid 3FTxs

From a cDNA library of *Sistrurus catenatus edwardsii* venom gland transcripts, we identified three singletons encoding 3FTxs [116]. Using a targeted approach, we performed reverse transcription polymerase chain reaction (RT-PCR) using forward primers encoding 3FTx signal peptide and sequenced 96 random clones to identify five 3FTxs expressed in the *S. catenatus edwardsii* library. All of them belong to non-conventional 3FTxs, with the fifth disulfide bridge in loop I. They contain 1–3 potential *N*-glycosylation sites. They show poor sequence identity and similarity to elapid and colubrid 3FTxs [116]. We determined the gene sequences for all five 3FTxs [117]. All these genes have three exons and two introns, similar to elapid and hydrophiid 3FTxs. The first exons encoding the signal peptide regions are highly conserved. Similarly, all segments in introns (when present) are highly conserved compared to other 3FTx genes. In contrast, exon segments are either highly conserved or distinctly different from the corresponding regions of other 3FTx isoforms [117]. As the insertion or deletion of segments defines evolutionary history, we proposed that some exon segments are exchanged with distinctly different ones during the evolution of these 3FTx genes. Such “switching” of segments in exons drastically alters the molecular surface and thus alters the molecular target of these 3FTxs [117]. This phenomenon of accelerated segment switch in exons to alter targeting (ASSET) may play an important role in the evolution of 3FTxs and other snake venom toxins [117,118]. No structural or functional details of these 3FTxs have been characterized.

Several 3FTxs have been identified in various viperid snakes, including Lachesis muta, Echis coloratus, Bothrops moojeni, Protobothrops flavoviridis, Crotalus adamanteus, Crotalus cerastes, Crotalus durissus terrificus, Crotalus viridis, Crotalus tigris, Azemiops feae, and Vipera transcaucasiana [119,120,121,122,123,124,125,126,127]. In general, 3FTxs are minor components in viperid venoms. Several of them are N-glycosylated, unlike typical elapid neurotoxins which rarely have posttranslational modifications. Interestingly, the signal peptides of viperid 3FTxs show high sequence identity and similarity to elapid, hydrophiid, and colubrid 3FTxs but share poor sequence identity and similarity in the mature toxin sequence. To our knowledge, none of the viperid 3FTxs have been characterized for their functions. We expect that these viperid 3FTxs to have distinct pharmacological profiles.

### 3.4. Acid-Sensing Ion Channels (ASICs) and Pain-Modifying Toxins

Acid-sensing ion channels (ASICs) are proton-gated Na^+^ channels activated by extracellular pH. Four genes encode six isoforms (1a, 1b, 2a, 2b, 3, and 4) that assemble to form these homo- and heterotrimeric voltage-insensitive channels with different pH sensitivities and pharmacological profiles [128,129,130,131,132]. ASICs are among the principal players in the pain pathway [128,132,133,134,135]. The first snake toxin that interacts with ASICs to be isolated was from the Texas coral snake (*Micrurus tener tener*). This toxin, named MitTx, is a heteromeric complex composed of Kunitz- and phospholipase-A_2_-like proteins and functions as a potent, persistent, and selective agonist activating peripheral ASICs in nociceptive neurons and evoking pain [136]. Here, we will not discuss MitTx further as 3FTx is not a part of this complex (for details on MitTx, see refs. [137,138]).

Dichot et al. described a new class of 3FTxs from black mamba (*Dendroaspis polylepis polylepis*) venom that inhibits ASICs expressed in central and peripheral neurons and abolishes pain [13]. They isolated two isoforms of 3FTxs and named them mambalgin-1 and mambalgin-2, which differ by one residue (Tyr4 is replaced by Phe4 in mambalgin-2; Thr23 is replaced by Ile23 in mambalgin-3, ref. [137]). They are potent, rapid, and reversible inhibitors of rat homomeric ASIC1a and heteromeric ASIC1a + ASIC2a or ASIC1a + ASIC2b channels, with a similar potency (IC_50_ values of 55 nM, 246 nM, and 61 nM, respectively) [13]. Mambalgins bind to the closed and/or inactivated state of the channels and modify their affinity for protons. Thus, they are gating modifiers. Mambalgin-1 decreases ASIC current amplitude to 13% of the control in central spinal cord neurons. They have no effect on ASIC2a, ASIC3, ASIC1a + ASIC3 and ASIC1b + ASIC3 channels, as well as on TRPV1, purinergic P2X2 receptor, serotonin (5-hydroxytryptamine) type 3A (5-HT_3A_) receptors, Na_v_1.8, voltage-gated calcium (Ca_v_) 3.2, and voltage-gated potassium (K_v_) 1.2 channels. They induce analgesic effects as potent as morphine in various animal models, but their effects are resistant to naloxone, with much less tolerance and without respiratory distress [13]. In ASIC1a knockout mice, mambalgins completely fail to show central analgesic effects, identifying ASIC1a as the main analgesic target. Mambalgins show in vitro analgesic effects through inhibition of homomeric ASIC1a and heteromeric ASIC1a + ASIC2b channels, while in vivo analgesic action is mainly targeted to neurons expressing ASIC1a + ASIC2a channels [13]. Mambalgins induce analgesic effects by targeting different subtypes of ASICs after local and central injections in models of acute and inflammatory pain. They reverse both mechanical and thermal inflammatory hyperalgesia after systemic i.v. administration. The peripheral ASIC1b- and systemic ASIC1a-containing channels participate in the antihyperalgesic effects of mambalgins [139]. After intravenous and central injection, mambalgins reduce neuropathic pain. In the central nervous system (CNS), involvement of different subtypes of ASICs can lead to opioid-dependent (ASIC1a and/or ASIC1a/ASIC2b channels) or opioid-independent (ASIC1a/ASIC2a channels) analgesia [13,140]. All three mambalgins were synthesized, and their three-dimensional structures were determined by NMR and X-ray crystallographic methods (PDB: NMR—2MFA and 2MJY; X-ray—5DU1, 5DZ5, and 5DO6 (T23A mutant)) [141,142,143,144]. The structures show similarity to those of other typical 3FTxs [141,142,143]. The short loops I and III flanking the longer loop II allow it to form a long antiparallel double-stranded sheet that protrudes extensively into the solvent. In the crystal structure, the central loops of two molecules form a β-sheet [143]. Interestingly, loop III has two distinct conformations in different crystal forms.

Mourier et al. also synthesized 10 Ala-substituted mutants (H21A, T23A, F27A, R28A, N29A, L30A, K31A, L32A, I33A, L34A, and K57A) and evaluated the wild-type (WT) and Ala mutants on ASICs heterologously expressed in *Xenopus* oocytes [143]. The WT mambalgin-1 potently inhibits homomeric rat ASIC1a, rat ASIC1b, and heteromeric rat ASIC1a + ASIC2a channels with IC_50_ values of 3.4, 22.2, and 152 nM, respectively. The Ala mutants H21A, T23A, N29A, L30A, K31A, and K57A show similar affinity to the WT toxin towards the ASIC1a channel. In contrast, F27A, R28A, L32A, I33A, and L34A show a significant increase in the IC_50_ values, suggesting their participation in ASIC1a binding. Interestingly, the L32A substitution results in a drastic three-order-of-magnitude reduction in affinity. Based on these results, Mourier et al. identified the key amino acid residues involved in the interaction with the ASIC1a channel [143]. Using double mutant analysis, they evaluated the proximity by studying the interactions of F27A, L32A, and L34A mutants with the ASIC1a-F350L mutant channel. The results suggest the proximity of Leu-32 in mambalgin-1 and Phe-350 in ASIC1a [143]. Based on these data, the authors refined the complex model of the interaction between mambalgin-1 and the rat ASIC1a channel protein. All three mambalgins inhibit ASICs with similar potency. Thus, substitution of residues at positions 4 and 23 does not contribute to the interaction with the ASIC.

Cryogenic electron microscopy (Cryo-EM) maps/structures of mambalgin-1 with chicken (Electron Microscopy Data Bank (EMDB) entries: EMD-6900, EMD-7296) and human ASIC1a channels (EMDB: EMD-30347; PDB: 7CFT) have been determined [14,145]. Each subunit resembles a hand (extracellular part) and a forearm (transmembrane). The individual domains in the hand are identified as the finger, thumb, knuckle, and palm [132]. The channel pore contains the channel gates and the selectivity filter lined by transmembrane α-helices, two from each of the three subunits. Three toxin molecules bind to the channel trimer. Each mambalgin-1 molecule interacts almost exclusively with a single ASIC1a subunit [145]. The complex structure shows that mambalgin-1 interacts directly with the extracellular thumb domain of chicken ASIC1a, rather than inserting into the acid-sensing pocket. The basic residues (His6/Lys8) at the tip of loop I, and those (Arg28/Lys31) of loop II, interact with the acidic regions of α4- and α5-helices of the thumb domain, respectively. The hydrophobic patch (Met25/Pro26/Phe27/Leu30/Leu32) in loop II also contributes to the interaction with the hydrophobic region in the α5-helix [145] (Figure 1e). The relocation of the thumb domain disrupts the acidic pocket, and through an allosteric mechanism, ASICs are inhibited. These interactions were also supported by site-directed mutation of several residues in ASIC1a. Chicken ASIC1 is pharmacologically quite different from human ASIC1a despite ~89% sequence identity. They exhibit different responses to mambalgin-1 [145]. Mambalgin-1 inhibits the full-length channels in a concentration-dependent manner (chicken vs. human IC_50_ values of 197.3 and 123.6 nM). At 500 nM, the decreases in channel activity were 60.4% and 19.6%, and at 10 μM the decreases were 78.9% and 31.9%, respectively. The trimeric human apo-ASIC1a^DC^ (60 C-terminal residues removed) shows a canonical chalice-like architecture (EMDB: EMD-30346; PDB: 7CFS) [14]. The ECD has a hand-like architecture with the palm, knuckle, finger, and thumb clasping a “ball” of β-strands. The apo-channel structure represents the resting state of hASIC1a [14]. Mambalgin-1 interacts with the thumb domain, as observed with the chicken ASIC1a channel. Psalmotoxin (PcTx1), a toxin from Trinidad tarantula (*Psalmopoeus cambridgei*) venom, binds to the thumb, palm, and β-ball domains of chicken ASIC1a, while MitTx interacts with the palm and thumb domains. Arg28 at the tip of loop II is stretched toward Asp347 and Asp351 of the α5-helix of the thumb domain [14]. The distances between the Cα atoms of Arg28-Asp347 and Arg28-Asp351 are 10.5 and 10.2 Å, respectively. Phe352 is nestled within a hydrophobic cluster (Met25, Phe27, Leu32, and Leu33) in loop II, mediating hydrophobic interactions. Mambalgin-1 forms multiple polar contacts with the channel through its loop I: Gln5 and His6 form hydrogen bonds with the side chain of Tyr360, and Lys8 forms salt bridges with Asp300 [14]. Loop III and the upper scaffold region have no contact with the channel. Q5A, H6A, K8A, F27A, and R28A mambalgin mutants exhibit reduced inhibition. Similarly, the toxin is less effective in inhibiting D347A, D351G, F352L, and Y360A mutants of human ASIC1a [14]. The transmembrane pore of the complex has a diameter of less than 2.0 Å in the gate, similar to a closed channel. The shifts of the thumb domain and transmembrane helices result in a less compact conformation than that of the apo form in the resting state, but the expanded conformation of the acid pocket and the closed pore are not altered. These conformational changes result in reduced proton sensitivity of ASIC1a. Mutational studies indicate that intrasubunit interactions play crucial roles in the activity modulation of ASICs from different species [14]. In the resting-state ASIC1a channel, the acidic pocket is expanded and the thumb domain is away from the central β-ball, finger, and palm domains. During activation and desensitization, the acidic pocket collapses, allowing the thumb and finger domains to approach and interact with each other. Mambalgin inhibits the channel by “proton sensor trapping”, locking it in a closed state and impeding the allostery-based channel modulation [14]. Similar mechanisms are utilized by various polypeptide toxins to modulate ion channel activity [146,147].

To understand the molecular details of the interaction between mambalgins and rat ASIC1a, Salinas et al. performed alanine-scanning analyses of loop I (tip: Q5, **H6,** and K8; core: V10, T11, H13, R14, and M16) and loop II (side: **N22** and M25) residues [148], in addition to previously described Ala mutants (loop II: H21, T23, **F27**, **R28**, N29, L30, K31, **L32**, **I33**, and **L34**; loop III: K57) of the toxin [143]. (The mutation residues that lead to more than 10-fold loss of function are shown in bold). They also studied the effects of site-directed mutations of the rat ASIC1a (α4-helix: D311A, R315A, **Y316A**, Y316F, E319A, **N320A**, and N322A; α5-helix: E342A, D345A, D349G, **F350L**, E353A, K354A, D355A, Q356A, E357A, **Y358A**, **Y358F**, and E362A) [148]. The residues in bold lead to more than 10-fold loss of activity. The tip of loop I, particularly H6, along with several residues in loop II (N22, F27, R28, L32, I33, and L34), is involved in the interaction with rat ASIC1a. Although F27A and R28A mutations result in significant loss of activity, F27Y and R28K mutants retain full activity. On the channel side, a cluster of four residues, two each in the α4-helix (Y316 and N320) and α5-helix (F350 and Y358), is central for the interaction with mambalgin [148]. The Y358F, but not Y316F, mutation strongly affected the inhibition. Three residues, E342, D345, and D349, in rat ASIC1a (equivalent to E343, D346, and D350 in chickens and E344, D347, and D351 in humans) are potential candidates to interact with the R28 residue of mambalgin. However, E342A, D345A, or D349G mutations do not affect the inhibition. D311 and E319 are potential candidates to interact with K8, but D311A or E319A mutations do not affect the inhibition. Using double mutant cycle analyses, they showed that L32 in loop II probably interacts with F350 in the α5-helix in the upper thumb domain of rat ASIC1a [143], while K8 in loop I probably interacts with Y358 of the channel [148]. The proposed model suggests that mambalgin-1 interacts with the hinge between the α4- and α5-helices in the thumb domain and locks it. Thus, it prevents motion and stabilizes the expanded shape of the acidic pocket and thus the closed state [148].

ASIC1a and ASIC1b are alternative splice forms encoded by a single gene. Mambalgins potently and fully inhibit ASIC1a activity without any effect on the pH dependence of activation and with a modest shift to more acidic values in the pH dependence of steady-state desensitization [149]. In contrast, they only partially and less potently inhibit rASIC1b, and this inhibition is pH-dependent, showing an acid shift of the activation curve and an alkaline shift of the steady-state desensitization [138]. Mambalgin-3 more potently inhibits rat ASIC1a than ASIC1b (IC_50_ values: 3.9 nM and 38.3 nM, respectively) [150]. It completely inhibits ASIC1a at saturating concentrations, while the inhibition of rASIC1b reaches only ~ 60%. The authors targeted non-conserved residues at the interface between the thumb and palm domains of the adjacent subunit and mutated the residue in ASIC1a to the corresponding residue from ASIC1b. The single mutants A82T, H173S, F174Y, and A178P show similar susceptibility to mambalgin-3 inhibition, whereas the mutants S83T, Q84E, R175C, and E177G are slightly less sensitive to inhibition. The quadruple mutant (SQRE) closely matches the inhibitory effects seen on ASIC1b. The rise and decay times of currents in ASIC1a are not affected, while both ASIC1b and the ASIC1a SQRE mutant show concentration-dependent decreases. To identify the differences in the pharmacophores involved in interactions with rat ASIC1a and rASIC1b, the authors evaluated 20 Ala mutants covering all three loops. The results show that six residues, H6, F27, R28, L32, I33, and L34, form the ASIC1a pharmacophore, while the ASIC1b pharmacophore comprises two additional residues (K8 and M16) along with the six-residue pharmacophore of ASIC1a. Thus, mambalgins bind to the two ASIC1 variants in subtly different orientations to stabilize distinct non-conducting states. The site-directed mutations indicated that the differential susceptibility of rat and human ASIC1a is due to the N291K substitution in the rat channel. The results suggest that a combination of binding and allosteric substitutions defines the difference in mambalgin inhibition of rat versus human ASIC1b channels. Interestingly, the mambalgin pharmacophore of human ASIC1b is more similar to that of rat ASIC1a than rat ASIC1b.

Cancer progression is supported by microenvironmental acidification. Cancer cells adapt to low pH through activation of pH sensors. ASICs mediate cancer cell migration and invasion [151,152,153]. Thus, inhibition of ASICs may contribute to therapeutic strategies for treating cancer. Mambalgin-2 interacts with ASIC1a and induces cell cycle arrest and apoptosis in glioma cells and inhibits growth and migration of metastatic melanoma cells and lung adenocarcinoma [154,155,156]. Lyukmanova et al. studied the mambalgin-2 interaction with the homotrimeric ASIC1a and heterotrimeric α-epithelial Na^+^ channel (ENaC)/ASIC1a/γ-ENaC by molecular dynamics (MD) simulations [157]. Intermolecular contact analysis shows that the toxin interaction sites at the primary ASIC1a(+) subunit are almost identical in the hetero- and homotrimeric channels. Mambalgin-2 interacts with the ASIC1a(+) subunit mostly through loop II, although loop I and “head” residues (H13–M16) also make some contacts [157]. The interacting interfaces for complementary subunits are different. The toxin interacts with ASIC1a(−) through a few hydrophobic contacts, while with γ-ENaC(−) it engages in both hydrophobic and electrostatic interactions (D15–K91). Mambalgin binds to the complementary subunits via the apex of its “head” in both complexes. Ala variants of the “head” residues show significant reduction in toxin activity on both α-ENaC/ASIC1a/γ-ENaC and ASIC1a channels. Thus, mambalgins also use their “head” residues in the interaction [157].

### 3.5. GABA_A_ Receptor Modulators

γ-Aminobutyric acid type A (GABA_A_) receptors, along with nAChRs, glycine (GlyR), and serotonin (5-HT) receptors, belong to the pentameric Cys-loop superfamily of ligand-gated ion channels [158]. They are found predominantly in the CNS, and they mediate inhibitory postsynaptic transmission by transporting Cl^−^ ions across the cell membrane. Distinct subunit isoforms (α1–6, β1–3, γ1–3, δ, ε, π, θ, and ρ1–3) with multiple structural arrangements form GABA_A_ receptors [159,160,161]. Each subunit contributes transmembrane domain 2 out of four transmembrane domains to create the axial Cl^−^ channel across the membrane [162]. GABA_A_ receptors are implicated in clinical pathologies including epilepsy, schizophrenia, and chronic pain [163,164]. A major fraction (~50% of the venom) from *Micrurus mipartitus* (Costa Rican coral snake) venom shows potent toxicity (intracerebroventricular (i.c.v.), LD_50_, 2 mg/kg) in the mouse brain. The animals show periods of reduced basal activity, followed by bursts of intense seizures. Rosso et al. purified two 64-residue 3FTxs, which differ by one amino acid: Arg33 (micrurotoxin 1, MmTX1) or His33 (micrurotoxin 2, MmTX2) [15]. Recombinant and synthetic MmTX1 and MmTX2 show slightly lower but similar symptoms and toxicity compared to the native toxin in mice. In competitive binding experiments, using ^125^I-rMmTX2 to RBS shows high-affinity binding of WT MmTx2 (Ki, 0.8 nM), rMmTx1 (Ki, 3.0 nM), and rMmTx2 (Ki, 3.7 nM) [15]. Substitution of His33Ser in rMmTx2 results in the loss of specific binding indicating (a) the importance of Arg/His33 at the tip of the central loop and that (b) rMmTX2 may bind to the receptors with similar orientation as when α-neurotoxins bind to nAChRs. As both rMmTX1 and rMmTX2 evoke seizures, they may bind to GABA_A_ receptors [15]. Since *M. mipartitus* feeds on vermiform species, which use GABA_A_ receptors for locomotion [165], the authors hypothesized that these toxins may play an important role in prey paralysis [15]. In *Caenorhabditis elegans*, the disruption of GABAergic neurotransmission induces simultaneous contractions of the body wall muscles [165]. Indeed, rMmTx1 induces paralysis in *C. elegans* upon topical application. rMmTX2 does not show competitive binding to GABA_A_-receptor-depleted RBS with ligands of nAChRs, mAChRs, GlyRs, glutamate receptors (GluRs), and acetylcholinesterase [15]. Competitive binding experiments with seven compounds that bind to diverse regions on the GABA_A_ receptor, including agonists, antagonists, and allosteric modulators, show that the competitive antagonist (gabazine: EC_50_, 0.2 μM), agonists (muscimol: EC_50_, 2.1 μM; GABA: EC_50_, 2.7 μM), and positive allosteric modulators (diazepam: EC_50_, 4.9 μM; pentobarbital: EC_50_, 4.9 μM) are effective in reducing ^125^I-MmTX2 binding, while the partial agonist isoguvacine (EC_50_, 275 μM) is not so effective [15]. Interestingly, the non-competitive channel blocker picrotoxin (PTX) from *Anamirta cocculus* (a tree from the Acanthaceae family) potentiates PTX (EC_50_, 3.7 μM) ^125^I-MmTX2 binding to RBS. These studies indicate that the GABA_A_ receptor is the main target for MmTXs. rMmTX1 increases the amplitude of the GABA_A_ receptor-mediated current elicited by muscimol. The α1β2γ2 GABA_A_ receptor, the most abundant GABA_A_ receptor in the hippocampus [166], was expressed in human embryonic kidney (HEK)-293 cells, and the effects of synthetic MmTX1 (sMmTX1) were evaluated. At low muscimol concentrations, 100 nM rMmTX1 potentiates GABA_A_ receptor-mediated currents, but this effect is lost at higher agonist concentrations. sMmTX1 (100 nM) potentiates the current mediated by 300 nM muscimol by 2.7-fold. MmTX1 and MmTX2 allosterically increase receptor affinity for the agonist, similar to benzodiazepines, thereby potentiating receptor opening and macroscopic desensitization [15]. Benzodiazepines have a fast off rate (seconds), while MmTX1 and MmTX2 bind tightly with a much slower off rate (t_1/2_ of ∼8 min). Alternatively, toxin-induced GABA_A_ receptor opening may trigger a hyperpolarization-activated excitatory conductance to generate rebound action potentials [167].

α-Cobratoxin, a potent antagonist of muscle-type and neuronal nAChRs, also inhibits the α1β3γ2 GABA_A_ receptor with an IC_50_ of 236 nM [168]. It also inhibits other subtypes with lower potency. Binding competition studies indicate that the toxin binding sites at the β/α interface overlap with the orthosteric sites. Long-chain neurotoxins, but not short-chain and non-conventional neurotoxins, inhibit GABA_A_ receptors [168]. The central loop of long-chain neurotoxin confers targeting selectivity. The short-chain neurotoxin with a swapped tip of loop II inhibits GABA_A_ receptors. A synthetic peptide based on the loop II tip inhibits the receptor. Molecular dynamics studies show that loop II (25–36 residues) binds stably at the site under loop C of the β3-subunit [168].

Cryo-EM structure shows that α-cobratoxin binds to the β–α interface at both GABA binding pockets located in the receptor’s ECD [169]. It docks perpendicularly to the cylindrical GABA_A_ receptor, inserting its three loops. The toxin’s Thr6 and Phe65 form van der Waals interactions with Val199 of the receptor loop C. Loop II inserts into the GABA binding pocket and interacts with loops B and C of β3, and loops D, E and F of α1-subunits, establishing the main contact zone below loop C [169]. Arg33 and Arg36 side chains straddle the aromatic side chain of loop C Phe200. Arg33 also stacks below the Tyr205 ring, and its backbone carbonyl likely forms a hydrogen bond with the Thr202 hydroxyl group [169]. As the α-subunit lacks the aromatic residue at the tip of loop C, the toxin does not bind at α–β interfaces (Figure 1f). Similarly, α-cobratoxin may not bind α–γ or γ–β interfaces in αβγ receptors but may bind to δ- and ρ-subunits as their loop C has this aromatic residue. This binding mode is similar to the α-cobratoxin complex with *Lymnaea stagnalis* AChBP and the α-bungarotoxin complex with muscle nAChRs [87,170].

### 3.6. Ω-Neurotoxins, a New Class of nAChR Antagonists

Oh9-1 isolated from *Ophiophagus hannah* venom was shown to irreversibly inhibit carbachol-induced muscle contraction with an IC_50_ of 88 nM [171]. Thus, Oh9-1 is only four-fold less potent than α-bungarotoxin. Interestingly, Oh9-1 has less than 33% sequence identity with α-neurotoxins. It has shorter loops I and III (five and three residues shorter, respectively) but an extra residue in loop II. Furthermore, the lack of almost all the functional site residues conserved in α-neurotoxins provided the impetus for its functional characterization and study of its interaction with nAChRs. We expressed recombinant Oh9-1 (rOh9-1) as a fusion protein with an extra N-terminal Met [35]. rOh9-1 shows potent, reversible postsynaptic neurotoxicity in CBCM preparations with an IC_50_ of 7.2 μM (carbachol-induced muscle contraction). The difference in potency could be ascribed to (i) undetectable amounts of high-affinity neurotoxins contaminating native Oh9-1 and/or (ii) the presence of an additional N-terminal Met. rOh9-1 shows selective inhibition of muscle-type rat α1β1εδ/α1β1γδ (adult/fetal) and neuronal rat α3β2 subtypes, with IC_50_ values of 3.1 μM, 5.6 μM, and 50.2 μM, respectively. It weakly inhibits human α3β2 and α3β4. It does not inhibit human neuronal α7, α9α10, α4β2, and α4β4, nor rat neuronal α4β2 subtypes, even at 100 μM. rOh9-1 does not inhibit other Cys-loop receptors, such as human α1β2γ2 and ρ1 GABA_A_ subtypes, at 30 μM. Interestingly, rOh9-1 induces concentration-independent potentiation of GlyRα1 receptors from 100 μM to <1 pM. To determine whether Oh9-1 is an allosteric or orthosteric antagonist, we examined the effect of serial concentrations of ACh on the rat α1β1δε subtype at 0, 3, and 10 μM rOh9-1. Oh9-1 shifts the ACh concentration–response curve to the right, without any change in the maximum response, indicating that Oh9-1 is a competitive antagonist of ACh, similar to α-neurotoxins. To delineate its functional site, we performed site-directed alanine scanning mutagenesis of 12 residues at the tip regions of three loops, except for P28 and P31. We evaluated the effects of 10 μM and 100 μM concentrations of 12 Ala variants on Ach-evoked currents of rat α1β1δε and α3β2 nAChR subtypes, respectively. The loop II residues, T23, T24, M25, F26, and F27, play critical roles in interaction with the rat α1β1δε receptor, and mutation of these residues leads to almost complete loss of activity. Mutation of H7, K22, H30, and K45 also leads to a substantial loss. Interestingly, mutation of N29 and V32 at the tip of the central loop had no impact on activity, while the Y46A substitution significantly enhanced activity. In Oh9-1-rat α3β2 interactions, a distinct set of residues are involved. M25A, F27A, and H30A show significant loss of activity. T23A and F26A show significant loss of inhibition with rat α1β1δε but have no effect on the interaction with α3β2. The T24A mutation leads to a significant loss of activity at α1β1δε but a gain at α3β2. The Y46A substitution increases activity at α3β2. Similar to α-neurotoxins, loop II residues play important roles in receptor interactions, but distinct Oh9-1 residues are involved in interactions with two receptor subtypes (α1β1δε and α3β2). Thus, Oh9-1 uses distinct but overlapping functional site residues to interact with two nAChR subtypes. Furthermore, the functional site residues in Oh9-1 involved in interactions with nAChRs are distinctly different from those in α-neurotoxins. Oh9-1 and related toxins share 3FTx folds with α-neurotoxins and bind orthosterically to the ACh binding pocket of nAChRs but use different functional site residues. Furthermore, this group of neurotoxins is phylogenetically distinct and has probably evolved independently from α-neurotoxins. Hence, this new group of neurotoxins was named Ω-neurotoxins. Thus, Ω-neurotoxins evolved through an unusual and unique case of functional convergence. Currently, we are evaluating the functional properties of other members of Ω-neurotoxins.

### 3.7. δ-Elapitoxins (Calliotoxin and Related Sodium Channel Activators)

*Calliophis bivirgatus* (blue coral snake) and *C. intestinalis* (banded Malayan coral snake) are Asian coral snakes with unusual long venom glands, extending up to one quarter of the length of their body. They feed on other snakes, including kraits and king cobras [172]. Takasaki et al. described some of the toxin components of *C. bivirgatus* (formerly *Maticora bivirgatus*) venom and purified two phospholipase A_2_ enzymes and four 3FTx-like proteins. They determined the N-terminal 41 residue sequence of maticotoxin A [173]. The toxin profile of *C. bivirgatus flaviceps* venom was determined by proteomic analysis [174]. Unlike neurotoxic snake venoms, which induce flaccid paralysis, *C. bivirgatus* venom uniquely induces spastic paralysis in CBCM preparations [172]. These large muscle contractions and fasciculations are reduced by the addition of the sodium channel antagonist tetrodotoxin. The toxin responsible for this unusual pharmacology was purified using activity-guided fractionation [172]. This toxin belongs to the 3FTx family and was named calliotoxin (δ-elapitoxin-Cb1a). Calliotoxin enhances peak inward current and delays inactivation of the human sodium channel Na_V_1.4 ectopically expressed in HEK-293 cells. Calliotoxin voltage-dependently delays the inactivation time constant and significantly increases inward ramp currents. This is the first Na_V_ activator isolated and purified from snake venom. The authors have discussed evolutionary advantages and the role of sodium channel activators in prey subjugation [172].

Dashevsky et al. elaborated the toxin profile of *C. bivirgatus* venom using transcriptomics and proteomics approaches, with a specific focus on 3FTxs [175]. The transcriptome contained 125 unique toxins, with 3FTx, Kunitz-type inhibitors, and phospholipase A_2_s as major venom components. The proteomics data strongly supported the presence of abundant toxins in the venom. They identified 67 contigs of 3FTxs. Most of these 3FTxs have yet to be characterized for their target receptor/ion channel/enzyme and function. They are clustered under several orphan groups. Tan et al. used the venom gland transcriptome to define the toxin profile of *C. bivirgatus flaviceps* venom [176]. They identified 74 transcripts and 20 3FTxs. The toxin profile of *C. intestinalis* venom was evaluated by proteomics. The authors identified 12 toxin families, with approximately 21% of total venom proteins being 3FTxs [177]. *C. intestinalis* venom inhibits Na_V_1.4 peak current without affecting the kinetics of fast inactivation, unlike calliotoxin isolated from *C. bivirgatus* venom [178]. Furthermore, *C. intestinalis* venom does not exhibit voltage dependence of activation or fast inactivation. The authors analyzed the venom proteome using the *C. bivirgatus* transcriptome as the reference. These differences could be due to: (a) the presence of calliotoxin-related toxins with subtle functional changes; (b) variable subtype selectivity towards NaV channels; (c) concentration-dependent function of toxins; and (d) the contribution of other toxins acting on the neuromuscular junction [178]. We have compiled the orphan toxin families using all 3FTx sequences from these publications. Molecular details of interactions between calliotoxin and related toxins with various sodium channels may help in understanding their distinct pharmacological profiles.

### 3.8. Potassium Channel Activator

Potassium channels play important roles in various biological processes, specifically in electrical signaling, and their malfunction is linked to cardiac and neuronal diseases and cancer [179,180,181,182,183]. As with other ion channels, potassium channels are regulated by activation (in response to stimuli such as ligand, voltage, and pH) and stimulus-independent inactivation from the activated state [184,185]. The pH-activated potassium channel of streptomyces A (KcsA) from *Streptomyces lividans* is an extensively studied potassium channel [186]. This was the first potassium channel whose three-dimensional structure was determined by X-ray crystallography. The structure provided important structural and mechanistic insights on the molecular basis for K^+^ ion selection and conduction. The structural platform helped in understanding mechanistic details of ion selectivity, channel gating by pH, and voltage-gated channel inactivation [186]. For this work, Roderick MacKinnon received the Nobel Prize in Chemistry in 2003. KcsA’s activation gate is controlled by a few cytosolic ionizable residues. At low pH, protonation leads to the loss of critical ionic interactions in the helical bundles, resulting in channel opening. Within 1–3 s of channel opening, slow (or C-type) inactivation occurs [187,188].

Rivera-Torres et al. used a direct toxin pull-down assay with immobilized KcsA to isolate a KcsA binding toxin from eastern green mamba (*Dendroaspis angusticeps*) venom [189]. Based on its theoretical mass, they named this 3FTx Tx7335. Subsequently, it was purified using retention time and mass-guided chromatographic methods. The amino acid sequence of Tx7335 shows unusually located Cys residues: C_1_-C_2_-C_3_C_4_-C_5_-C_6_C_7_-C_8_, instead of the typical 3FTx arrangement of C_1_-C_2_-C_3_-C_4_-C_5_-C_6_C_7_-C_8_. Thus, the disulfide bridges in Tx7335 are expected to be different. The Cys residues in 3FTx typically form four disulfide bridges: C_1_-C_3_, C_2_-C_4_, C_5_-C_6_, and C_7_-C_8_. Despite the systematic efforts, Rivera-Torres et al. identified only one disulfide bridge, C_2_-C_5_, and this S-S bridge does not exist in typical 3FTxs [189]. The authors expected major disruptions in the structure due to the spatial proximity of these two Cys residues. Single-channel measurements show that Tx7335 increases channel openings of reconstituted KcsA in planar lipid bilayers in a dose-dependent manner. Tx7335 (2.0 μM) induces an ~8-fold increase in mean open times for KcsA channels [189]. The authors postulated that toxin binding to the extracellular turret region induces conformational or dynamic changes that are allosterically coupled to the selectivity filter. So far, we have not identified another 3FTx with similar Cys residues. It will be interesting to determine its structure and the molecular details of interactions with KcsA.

### 3.9. αδ-Bungarotoxins

Utkin et al. isolated and characterized a novel group of long-chain α-neurotoxins that show differential affinity towards the two agonist binding sites of muscle-type nAChRs [190]. They are more active at the α–δ interface. They identified three isoforms (αδ-BgTx-1–3) in Malayan krait (*Bungarus candidus*) genomic DNA analysis and isolated two isoforms (αδ-BgTx-1 and 2) from its venom. The toxins are structurally similar to α-bungarotoxin with 73 amino acid residues and five disulfide bridges [190]. Their toxicity in mice is similar to that of α-bungarotoxin. In CBCM preparations, αδ-BgTx-1 shows potent but reversible postsynaptic effects. It binds to α7 nAChRs with high affinity. Interestingly, αδ-BgTxs discriminate the two binding sites in the *Torpedo californica* and mouse muscle-type nAChRs [190]. They show up to two orders of magnitude higher affinity for the α–δ site as compared to α–ε or α–γ binding site interfaces. The authors proposed that the lower number of positively charged residues may be responsible for the observed functional difference in αδ-BgTxs [190]. The αδ-BgTxs provide new tools for studying nAChRs. The studies elegantly show that minor changes in some amino acid residues result in altered interface selectivity [191]. Similarly, single point mutants of short-chain α-neurotoxins can also be used to study interface selectivity for nAChRs [192,193].

Agonists such as ACh (endogenous) and nicotine (exogenous) bind at the interface between adjacent subunits. These sites are formed by six non-contiguous regions (loops A–F), three loops each from both subunits. The α-subunit, the principal (+) component, contributes three loops (A–C) of highly conserved residues, while the complementary (–) component, mostly a non-α-subunit, contributes three loops (D–F) that have lower levels of sequence conservation [194]. Thus, the two components of the agonist binding sites are non-equivalent, and the loops contribute differently to receptor function [195]. Several animal toxins bind to these agonist binding sites and orthosterically compete with the agonist to block neurotransmission by various nAChR subtypes. The distinct agonist binding interfaces define affinities and selectivity towards various agonists and antagonists [196]. To develop highly selective research tools and therapeutic agents, we need to consider subunit composition and their interfaces, localization, compartmentalization, and accessibility. αδ-BgTxs show that subtle changes in structure lead to variation in interface selectivity [190,191]. The data provide added motivation to re-evaluate molecular details of pharmacological properties of 3FTxs with careful consideration towards subtype, interface, and species selectivity. These studies provide us with great opportunities to delineate pharmacophores and design new and highly selective antagonists.

### 3.10. Σ-Neurotoxins

In our lab, we characterized the first member of a new class of neurotoxins, fulditoxin (fulvius dimeric toxin), from coral snake (*Micrurus fulvius fulvius*) venom [33]. Its potency for postsynaptic neuromuscular blockade of CBCM is comparable to Types I and II α-neurotoxins, but this neuromuscular blockade is completely reversible. Fulditoxin blocks cloned rodent and human α1β1δε nAChRs with 100-fold lower affinity compared to avian muscle-type nAChR [33]. It also blocks cloned human neuronal α7, α4β2, and α3β2 nAChRs, unlike short-chain neurotoxins that bind only to muscle-type nAChRs. Despite the absence of the functional residues of either canonical short-chain α-neurotoxins [60,61,197] or Ω-neurotoxins [35], fulditoxin competes with ACh binding in nAChRs, similar to α-neurotoxins and Ω-neurotoxins [33]. As of now, the amino acid residues involved in binding to nAChRs are not known. Interestingly, fulditoxin exhibits an unusual dimeric structure. Loop II of both fulditoxin subunits curl and interact with each other, in contrast to other non-covalent 3FTx dimers in which antiparallel β-sheets are formed between loop III of two monomers. The loop II His binds Zn^2+^ ions, forming a tetramer of dimers [33]. Based on its distinct dimeric structure and absence of typical functional site residues, we named this new class of nAChR-targeting neurotoxins Σ-neurotoxins. Several toxins show high identity with fulditoxin and have retained most of the residues involved in dimerization. Such *Micrurus* 3FTxs also belong to Σ-neurotoxins. They are classified into three classes, and the first two classes into three groups each, based on the presence or absence of the Zn^2+^ binding His residue and the C-terminal residues (for details, see ref. [33]).

## 4. Omics Technologies in the Discovery of 3FTxs

Before the age of omics, individual toxins were purified one at a time and characterized for their structure and functional properties. These studies dictated the need for significantly large amounts of venom. Thus, research focused on snakes that produced copious amounts of venom. This made it difficult to obtain data from low-venom-yielding species. Tremendous improvements in next-generation sequencing (NGS) techniques, transcriptomics, and proteomics (bottom-up and top-down), combined with significant reduction in costs for compiling venom toxin profiles, led to a data explosion and the discovery of a large number of 3FTxs [198]. For example, low venom yields in colubrid [199] and coral [200] snakes make it difficult to determine the toxin profiles of such venoms. Using advanced strategies, toxin profiles have been documented in several snake venoms [91,201,202,203,204,205,206,207,208,209,210,211]. As a significant number of toxins are relatively small-molecular-weight proteins, bottom-up and top-down proteomic approaches have been used to determine the toxin profiles of several venoms [212,213,214,215,216,217,218,219,220,221]. Through such high-throughput studies, even 3FTxs found in minute quantities have been identified in snake venoms. As many as 67, 55, 54, and 47 3FTxs have been identified in *Calliophis bivirgatus*, *Micrurus alleni*, *M. nigrocinctus*, and *M. mosquitensis*, respectively [175,221]. Surprisingly, the rattlesnake *Crotalus adamanteus* genome has 35 genes that could encode 3FTxs [127]. Thus, the recent explosion in the number of toxin-like protein sequences poses distinct challenges for structural and functional characterization. AlphaFold3 has provided an excellent predictive method to define the three-dimensional structure of 3FTxs [222]. Although common databases include such predicted models, these protein models often include the signal peptide regions (which are removed during protein maturation). This necessitates the resubmission of mature protein sequences for AlphaFold prediction to obtain the “correct” three-dimensional structures.

## 5. Identification of New Orphan 3FTxs

**Figure 3:**
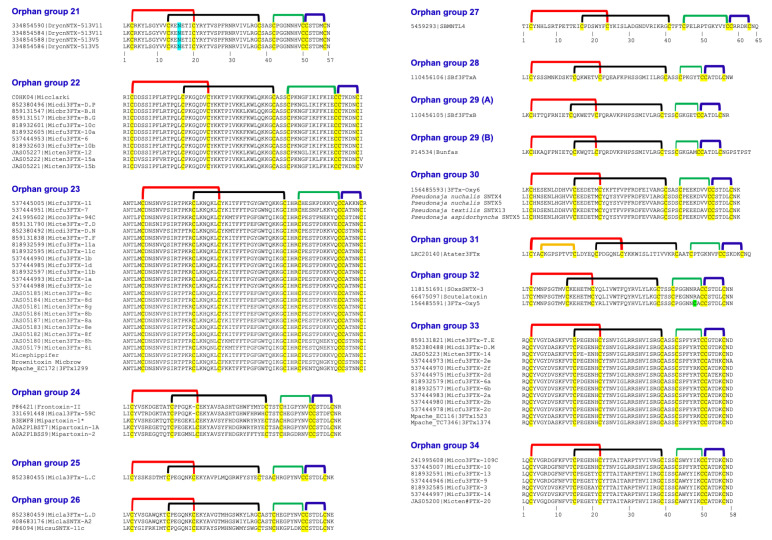

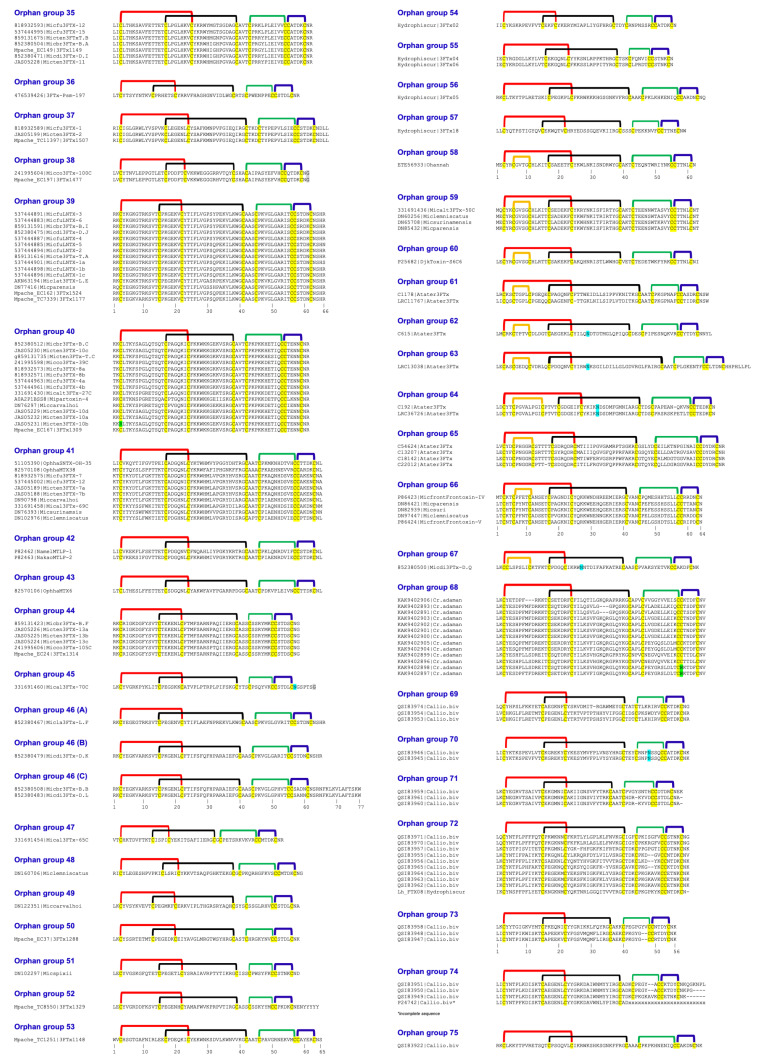

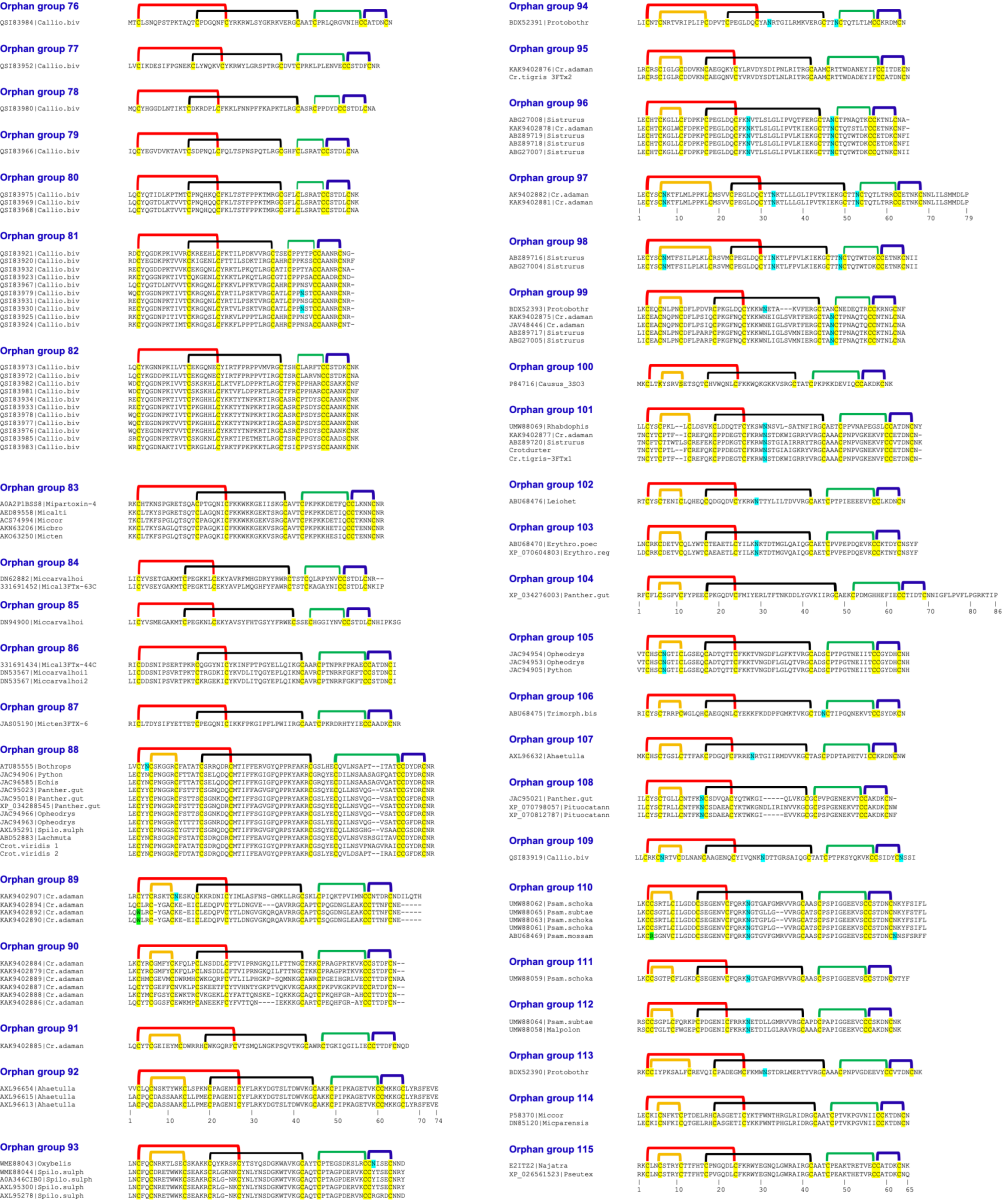
Multiple sequence alignment of 95 new groups of orphan 3FTxs. Typically, these orphan 3FTxs show low identity with other well-established classes of 3FTxs. These 95 groups of orphan 3FTxs have typical N-terminal and disulfide pairing framework. NCBI or UniProt accession numbers are indicated. Cys residues are highlighted in yellow, with disulfide bridges indicated above. The first disulfide bridge (S-S) is colored red, the second S-S is colored black, the third S-S is colored green, the fourth S-S is colored blue, the fifth S-S is colored orange. Predicted N-linked glycosylation sites (Ans-Xaa-Ser/Thr) are highlighted in cyan. Green highlights indicate unexpected mutations. Grey highlights indicate potential amidation of the C-terminal tail.

Currently, the prediction of protein function is still in its infancy. We analyzed the sequences of over 550 3FTxs, taking into account almost all 3FTxs whose pharmacological functions and target receptors/ion channels/enzymes are not known. We added a few new toxins to the previously identified 20 orphan groups of toxins (Figure 2, Table 1). Furthermore, we classified over 450 3FTxs into 137 groups of orphan toxins (Table 2). Sequences were curated from the NCBI and UniProt databases. Multiple alignments were performed using ClustalW, followed by manual adjustments. In general, 3FTxs within a group share high sequence identity and similarity (more than 75%). Typically, these orphan 3FTxs show poor identity with other well-established classes of 3FTxs, such as neurotoxins that target nAChRs (including classical α-neurotoxins, κ-bungarotoxins, Type III neurotoxins, and non-conventional neurotoxins), as well as those that target mAChRs, cardiotoxins/cytotoxins, platelet aggregation inhibitors, acetylcholinesterase inhibitors, L-type calcium channel inhibitors, and synergistic toxins. This also includes recently characterized nAChR antagonists (colubrid monomeric and dimeric neurotoxins, Ω-neurotoxins, Σ-neurotoxins, and αδ-neurotoxins), modulators of GABA_A_ receptors, α- and β-adrenoceptor modulators, ASIC modulators (mambalgins), and activators of sodium (δ-elapitoxin, calliotoxin) and potassium (Tx7335) channels. Figure 3 depicts the alignment of 95 groups of these 3FTxs with the typical N-terminal and disulfide pairing framework. Table 2 shows that most orphan 3FTxs retain the canonical framework, i.e., two-residue N-terminus before the first cysteine, four conserved disulfides with loop II generally longest, a three-residue interring gap being most common, and only a subset carrying a fifth disulfide in loop I or II. Diversity arises from defined minorities: N-terminal length variants (one, three, or four residues), distinct disulfide frameworks (including rare loss/uncertainty of the fourth S–S), “spare” cysteines that may or may not enable dimerization, unusually long C-terminal tails, and several clades where loop I approaches or exceeds loop II in length, implying alternative target-recognition geometries.

### 5.1. N-Terminal Variants

**Figure 4:**
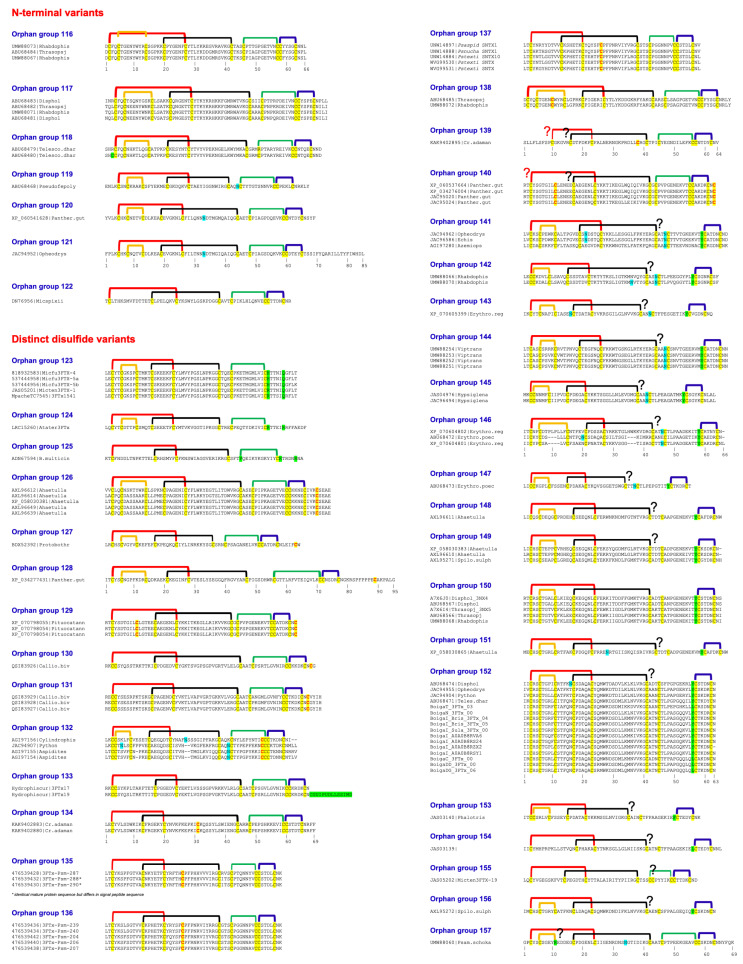
Multiple sequence alignment of the 7 new groups of orphan 3FTxs with variable numbers of N-terminal amino acid residues and 35 new groups of orphan 3FTxs with atypical disulfide pairing frameworks. Typically, these orphan 3FTxs show low identity with other well-established classes of 3FTxs. Cys residues are highlighted in yellow, with disulfide bridges indicated above. The first disulfide bridge (S-S) is colored red, the second S-S is colored black, the third S-S is colored green, the fourth S-S is colored blue, the fifth S-S is colored orange. Spare Cys residuesthat may or may not form disulfide bridge is colored red. Some of the disulfide pairing frameworks are uncertain, indicated by “?”. Predicted N-linked glycosylation sites (Asn-Xaa-Ser/Thr) are highlighted in cyan. Green highlights indicate unexpected mutations.

Most 3FTxs have two amino acid residues preceding the first Cys residue. Most colubrid toxins have a seven-residue extension [31,82,86]. A small number of colubrid toxins show unusual variations in the propeptide region and the N-terminal segments (for details, see ref. [36]). Some of the orphan 3FTxs have only one (orphan groups 116 and 122), three (orphan groups 117 and 118), or four (orphan groups 119, 120, and 121) residues (Figure 4, Table 2). A shorter N-terminal segment in 3FTxs before the disulfide bridge may protect the protein from degradation by aminopeptidases [223]. Longer N-terminal segments in colubrid 3FTxs may be protected by cyclization of Gln to pyroglutamate, which prevents protein degradation by aminopeptidase [224]. There are also colubrid 3FTxs without the pyroglutamate; in these proteins, the N-terminal segment is potentially protected by the presence of single or double Pro residues. These N-terminal Pro residues, like pyroglutamate, may protect the toxin protein from degradation by aminopeptidases via steric hindrance [224,225]. The impact of longer N-terminal segments on the function of 3FTxs is not yet clear. Regardless, all N-terminal variations appear to have evolved potential mechanisms to protect 3FTxs against aminopeptidase degradation.

### 5.2. Disulfide Bridge Variants

All 3FTxs have four conserved disulfide bridges (category I). There are two main categories of 3FTxs that have an additional fifth disulfide bridge; it is located at the tip of loop II in long-chain neurotoxins (category II) or at the tip of loop I in non-conventional neurotoxins (category III) [3]. Only 8 3FTxs out of over 1800 3FTxs (<0.5%, orphan 3FTx groups 123, 124, and 125) lack the fourth disulfide bridge due to the absence of both Cys residues (Figure 4). In these cases, Cys residues are replaced by Arg (single transition mutation from TGC/T encoding Cys to CGC/T encoding Arg), His (single transition mutation from CGC/T encoding Arg to CAC/T encoding His), or Gln (single transversion mutation from CAC/T encoding His to CAA/G encoding Gln) residues. Thus, it appears mutations lead from Cys → Arg → His → Gln. It is difficult to imagine so many substitutions occurring within two codons separated by 12 nucleotides. This loss of the fourth disulfide bridge may not affect the overall structure of 3FTxs. However, the C-terminal tails in these toxins are probably more flexible.

### 5.3. Additional Spare Cys Residues

Several short-chain neurotoxins isolated from the venoms of sea snakes and Australian snakes have one spare Cys residue (Cys4). The thiol group of this Cys is free [226,227]. This Cys is buried within the core of the 3FTx.

The free thiols are involved in covalent dimer formation in colubrid dimeric neurotoxins and homo- and heterodimeric synergistic toxins isolated from *Dendroaspis* venoms [86,228]. In colubrids, heterodimers are held together by a disulfide bridge formed between the free Cys residue in loop II of subunit A and the free Cys residue in loop I of subunit B [31]. In synergistic toxins, homo- or heterodimers are held together by a disulfide bridge formed between the free Cys residue in loop III of the subunits [228]. Thus, free Cys residues may drive homo- or heterodimerization and provide possibilities for exploring distinct target specificity and pharmacology.

We have identified five orphan groups of toxins (orphan groups 126 to 131) that have a spare Cys residue at the C-terminal end after the fourth S-S ring (Figure 4). There are five isoforms in *Ahaetulla prasina* and four isoforms in *Calliophis bivirgatus*. In these cases, they may form homo- or heterodimers among the 3FTx isoforms. There is only one 3FTx isoform in *Protobothrops flavoviridis* and *Pantherophis guttatus*, so they may form only homodimers. Alternatively, all the above 3FTx isoforms may form heterodimers with toxins from non-3FTx families.

There are also 3FTx isoforms that have a spare Cys residue in loop III (orphan group 132), loop II (orphan groups 134 to 137), or loop I (orphan group 138). These spare Cys residues may contribute to the formation of homo- or heterodimers, as described above. In orphan toxin group 139, toxins have a spare Cys residue in the C-terminal as well as another spare Cys residue in loop I. Whether these two Cys residues form an intrachain disulfide bridge between them, or form homo- or heterodimers, is unclear. There are 13 orphan toxin groups (140 to 152) that have one of the Cys residues (typically involved in the third S-S ring) mutated to Tyr (TGC/T to TAC/T; in orphan groups 140 to 150), Phe (TGC/T to TTC/T; orphan group 151), or Leu (TGC/T to TTA/G or CTC/T; orphan group 151). The Cys-to-Tyr and Cys-to-Phe substitutions are due to a single nucleotide mutation, while Cys-to-Leu substitution requires two mutations. It is difficult to predict the correct disulfide bridges in these toxins and whether the spare Cys residue is involved in dimerization.

## 6. Fascinating Three-Ring Circus in 3FTxs

**Figure 5:**
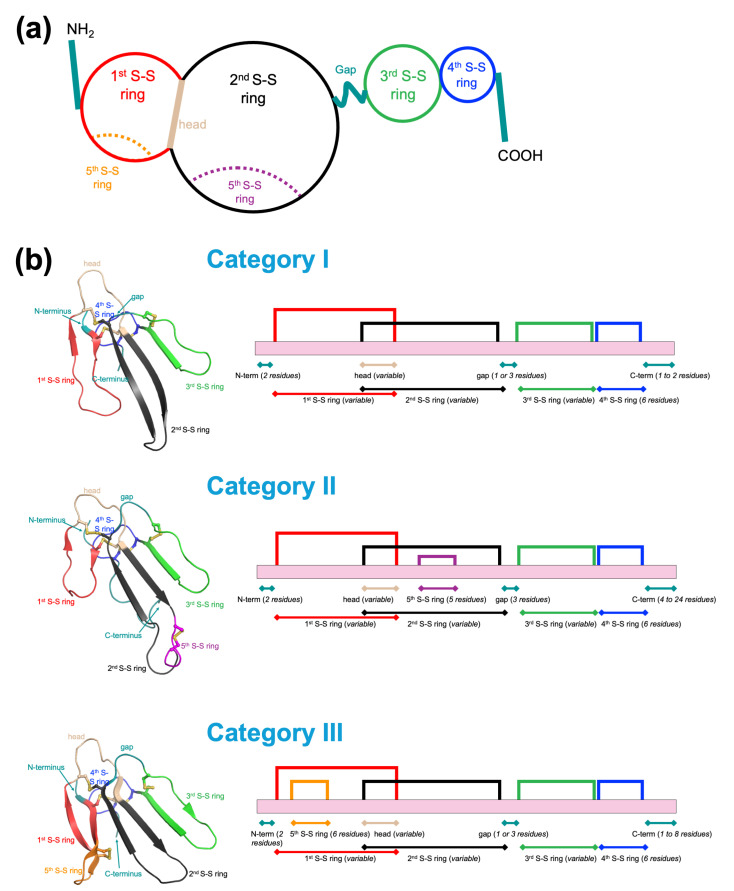
Cartoon depicting the topology of 3FTxs. (**a**) With the four conserved, signature S-S rings, the first (red) and second (black) rings are separated from the third (green) and fourth (blue) rings through a short gap (teal). For some 3FTxs, a fifth ring may also be present within the first or second ring. The overlapping segment between the first and second S-S rings is identified as the “head” segment (wheat). (**b**) Three general categories of structural 3FTx architecture. Three-dimensional structures of prototypical 3FTx in each category are depicted in cartoon models and color traced accordingly. Schematic representation is also depicted. Category I 3FTxs, represented here by erabutoxin (PDB: 1QKD), have the typical four disulfide pairing framework. Category II 3FTxs, represented here by α-cobratoxin (PDB: 2CTX), have an additional fifth S-S ring in loop II. Category III 3FTxs, represented here by bucandin (PDB: 1F94), have an additional fifth S-S ring in loop I. The numbers of amino acid residues in each structural element depicted here are indicated in the brackets and are summarized in Table 2.

3FTxs have four conserved disulfide bridges that create four Cys disulfide (S-S) rings (Figure 5a). Of these, the first three S-S rings manifest as variable-sized loops I, II, and III, while the fourth S-S ring is always a six-membered ring. In the three-dimensional structure, all four conserved disulfide bridges are located in the “core” region (palm), and the three β-sheeted loops dangle as “fingers”. In most 3FTxs, the N-terminal segment ahead of the first Cys has two amino acid residues. Colubrid neurotoxins provide glaring exceptions; most of them have six- or seven-residue N-terminal extensions. Some other rare exceptions are found among the orphan toxins described here (see above). The C-terminal segment after the last Cys of the fourth S-S ring tends to have variable length, although in the majority of cases it is short. The first two S-S rings are fused together with a short segment shared between them. This overlapping segment between the first and second S-S rings is located at the top of the “core” and, at times, is identified as the “head” segment. These first two S-S rings are separated by a short “gap” composed of either one (usually Gly) or three amino acid residues with a few exceptions. This Gly residue allows flexibility that could afford reorientation of the two pairs of S-S rings. When the gap is composed of three residues, at least one is capable of inducing turns in proteins, such as Ser, Thr, Asn, and Asp [229,230]. At times, these residues are substituted by Gly (allows the most flexibility) or Pro (induces a strong conformational constraint but affords cis–trans peptide bond configuration) [231]. Extremely rarely, the gap does not contain any of these six residues. We propose that the flexibility or rigidity provided by this gap may define the selective binding to the target.

In general, the loops (“fingers”) play important roles in binding to a specific target receptor, ion channel, or enzyme and define the pharmacology of 3FTxs. Loop II, commonly the longest of the three loops, has the greatest reach. Thus, loop II appears to play a key role in binding to the target. The other two loops may contribute depending on their length and reach. In several cases among the orphan toxins, loop I is almost equal in length (Table 2, denoted with ^) or even slightly longer compared to the respective loop II (for example, orphan groups 88 and 109) (Table 2). (Note: A difference of 1–2 amino acid residues in β-sheeted loops alters the length, but the width of loop tips may bring them within similar reach.) In these cases, either loop I or II will play a critical role in binding and recognition of the target. Although loop III in most 3FTxs is shorter, it also plays a prominent role in the function. For example, dendroaspin and mambin, which contain the RGD tripeptide sequence, are involved in binding to αIIbβ3 and inhibiting platelet aggregation [16,232], while the TAMW tetrapeptide sequence in calciseptine (and FS2) is involved in binding to the L-type calcium receptor and lowers the blood pressure [11,233] (Figure 1d). Thus, all three main loops contribute strongly to target recognition and binding.

The fourth S-S ring always contains one, two, three, or four turn-inducing amino acid residues, such as Ser, Thr, Asn, Asp, Gly, and/or Pro. Thus, disulfide bridges are easily formed due to these turn-inducing residues and the shorter distance between the Cys residues. This disulfide bridge also brings the C-terminal end closer to the N-terminal of the 3FTx. Therefore, this S-S ring is highly conserved and closely associated with the third S-S ring. The sequence CCXXXXC (where one or more X residues induce protein turns) is the typical landmark identifier for 3FTxs. In only a small number of 3FTxs is this fourth S-S ring absent (see above).

Category II and III 3FTxs have an additional fifth S-S ring in loops II and I, respectively (Figure 5b). These disulfide bridges introduce a “pinch” in the respective loops. These covalent links help extend the reach of these loops, much like the “pinch” pushes the air further in a tubular balloon. They also reduce the widening of the loop tips. In long-chain neurotoxins (category II), the fifth disulfide always creates a five-membered S-S ring. Invariably, one or more amino acid residues in this S-S ring are Ser, Thr, Asn, Asp, and Gly, which are capable of inducing turns in the protein structure. In long-chain neurotoxins, two of the three amino acid residues (Asp/Asn-Xaa-aromatic residue) are key components of the functional site and contribute to neurotoxin binding to nAChRs. Notably, Asp and Asn residues also induce turns. Substitution of these functional site residues leads to loss of neurotoxicity. Removal of the disulfide bridge leads to loss of binding to α7 nAChR but not to *Torpedo* nAChR. Thus, the fifth S-S ring contributes to binding to the novel target and, hence, to new function. In three long-chain toxins from *Hemiaspis signata* venom, this S-S ring contains Gly-Ser-Met. The consequence of such a drastic change on toxin function has not yet been documented.

The fifth disulfide bridge in category III (non-conventional toxins) is located in loop I. It most often forms a six-membered S-S ring. As expected, the intervening residues induce turns in the protein structure. In a significant number of toxins (65–70%), Pro plays an important role in inducing the turn and facilitating disulfide formation. The hydrophobic sulfur atoms of the disulfide bridge, along with the hydrophobic Pro (side-chain ring), probably tend to splay the tip of loop I away from the body of 3FTx.

As mentioned above, the three loops created by the first to third S-S rings play a vital role in molecular recognition of the target. At times, residues from the “head” (as in the case of mambalgin binding to α-ENaC/ASIC1a/γ-ENaC channel trimer) [157] or from the fourth ring (as in the case of ringhalexin-related toxins binding to the TF-FVIIa complex) [20] are involved in the binding to the target. Thus, it is imperative to evaluate the role of the entire molecular surface of 3FTxs, not just the three loops.

As with other toxins, 3FTxs are evolving at a relatively rapid pace, aided by positive Darwinian selection. These mutation and natural selection processes are affecting all three rings simultaneously. It is like a three-ring circus, where simultaneous performances are occurring in all three rings at a frenetic pace. For the first-time visitor to the circus, it appears to be a disorganized spectacle. In contrast, the changes in the three S-S rings of 3FTxs provide an exciting, intriguing, and engrossing challenge to understand and resolve a complex, yet subtle, interplay in molecular recognition between the toxin and its respective target. In addition to the three rings, the fourth and fifth S-S rings, as well as potential quaternary structures, enhance the challenge of deciphering their structure–function relationships.

## 7. Conclusions

Snake venoms are complex mixture of potent toxins, which are involved in immobilizing, killing, and digesting prey organisms. Thus, snake venom toxins have evolved through multiple duplication events followed by accelerated evolution and natural selection to target specific receptors/ion channels/enzymes [234]. Among various superfamilies of protein toxins, 3FTxs are one of the largest families of non-enzymatic proteins [3]. In recent years, we have collected sequences of a large number of 3FTxs with the advent of various transcriptomic, proteomic, and genomic technologies. Within the 3FTx family, neurotoxins are the largest group of well-studied 3FTxs. We recently analyzed over 700 amino acid sequences of neurotoxins that interact with nAChRs and classified them into over 150 distinct subgroups based on variations in their functional site residues [36]. Based on the interaction of various loops of cytotoxins with lipid membranes, 90 toxins were classified into eight groups (the second group is empty) [235]. Previously, we identified 20 clades of orphan 3FTxs whose pharmacological target proteins were not yet identified [2]. In the last two decades, the structure and function of ten out of twenty groups have been characterized. In addition, several new classes of 3FTxs, including colubrid neurotoxins, β-cardiotoxin, α-adrenoceptor inhibitors, modulators of GABA_A_ receptors and ASICs, new classes of nAChR antagonists (Ω-, αδ- and Σ-neurotoxins), and activators of sodium and potassium channels, have been characterized. Here, we analyzed over 550 3FTxs and classified them into 157 orphan groups. Most 3FTxs are small, with 52 to 70 amino acid residues. They can be easily synthesized using peptide chemistry, either directly or through chemical ligation methods [86,141,142,143,144]. These methods could facilitate the inclusion of unnatural amino acid residues if needed to improve the affinity and/or selectivity of 3FTxs. They can also be recombinantly expressed in large amounts [236]. It is important to note that some of these 3FTxs have potential N-glycosylation sites. If these glycosylation moieties play a crucial role in function, one needs to consider the recombinant expression of such 3FTxs in mammalian or insect cell lines.

Although 3FTxs share a similar overall protein scaffold, subtle conformational differences appear to play important roles in the recognition of specific target proteins. Therefore, it is imperative that the three-dimensional structures of 3FTxs be determined. At times, quaternary structures may exist in some 3FTxs. These novel toxins may have interesting pharmacological properties and distinct molecular targets, thereby allowing their use as investigational tools. Thus, the functional characterization and identification of target proteins of these orphan 3FTxs are critical first steps. There are several automated, high-throughput methods to enable in vitro characterization of 3FTxs. For example, imaging-based assays for intracellular Ca^2+^ or pH changes [237], automated patch clamp [238], affinity-based microarrays or mass spectrometry assays [239,240,241], thermal stability-based methods [242], and many other approaches can be utilized towards gaining an understanding of new protein targets, deciphering novel pharmacophores, and development of novel research tools, as well as diagnostic and therapeutic agents.

## Figures and Tables

**Table 1:** The twenty groups of orphan 3FTxs as initially classified in 2003 [2]. UniProt or NCBI accession numbers are listed. Accession numbers in bold are new sequences that can be added to the groups since it was first published. Some 3FTxs that have been named and characterized in detail, with names appearing in the brackets.

Group	Toxins
Orphan group I	Q9W717 (natralexin); **C0HJT5** (ringhalexin)**; ETE58964** (ophiolexin), **APB88857** (najalexin)
Orphan group II	O93422, Q9YGI2, Q9YGI1, P29180, P01401, P01400, P29179, P01399, P25680, P29181, P29182, Q9YGI4, Q9W7I3, O42256, O42255, P25679, P82935, **ADN67577**, **P85520**, **Q802B2**, **ADF50020**, **ADF50022**, **ADF50021**, **C1IC49**, **Q53B61**, **A2CKF7**, **Q2VBN2**, **Q802B3**, **Q8AY51** (bungatoxin)
Orphan group III	Q9YGI8, P83346 (bucain), **ADF50015**, **P0C554**
Orphan group IV	P15818 (Q9YGI9), P81783 (candoxin), **CAA06886**, **ADF50017**, **ADF50016**, **ADF50019**
Orphan group V	Q9YGJ0 (γ-bungarotoxin; AAD41806), O12963, Q9YGH9
Orphan group VI	P25676, P01415
Orphan group VII	P43445
Orphan group VIII	Q9W727, Q9DEQ3, P82464, **A8N286** (haditoxin)
Orphan group IX	Q9YGI0, P79688, **Q8AY53**, **Q800Y3**, **Q8AY52**, **ADN67594**
Orphan group X	P18329
Orphan group XI	P01406, P01405, P01404
Orphan group XII	Q9PRI1, Q9PUB7, **AKM28629**, **JAS05198**, **JC7187**, **Mpache_TC7570|3FTx1617**
Orphan group XIII	P19004
Orphan group XIV	P24778, **P49122**
Orphan group XV	Q91996, P62375 *, Q91126, Q91137, Q9W716, P14541, P62394 *, P49122, P01473, P01474, **P62377**, **P0DUK7**, **P62390**
Orphan group XVI	P19003
Orphan group XVII	Q9YGH0, Q9PW19, bulongin, **Q8JFX7**
Orphan group XVIII	P14534, **P0C552**
Orphan group XIX	P81782 (bucandin), P25682, **ETE56933**, **C0HKZ8** (actiflagelin)
Orphan group XX	P25677, P25678, **770503B**

* Accession numbers changed since they were first published.

**Table 2:** Number of amino acid residues in each structural feature in 3FTxs for different orphan groups. Colors of disulfide bridges (S-S) correspond to color scheme used in other figures. For groups with distinct disulfide variants (groups 123 to 157), some of the disulfide pairing frameworks are uncertain; hence, the number of residues in the relevant loops is indicated with “?”.

Orphan 3FTx Group	N-term	First S-S (Fifth S-S)	Second S-S	Gap Residues	Third S-S	Fourth S-S	C-Term
20 groups of orphan 3FTxs initially classified in 2003 [2]							
I	2	22	26	3	12	6	2
II	2	22 (6 *)	24–26	3	12	6	2
III	2	22	26	3	13	6	2
IV	2	24 ^ (6 *)	25	3	13	6	1–2
V	2	22 (6 *)	29	3	12	6	2
VI	2	19	24	3	13	6	2
VII	2	22	25	3	9	6	2
VIII	2	22	25	3	13	6	2
IX	2	22	25	3	13	6	2
X	2	22	25	3	13	6	2
XI	2	20	23	1	12	6	1
XII	2	20	23	1	12–13	6	2
XIII	2	19^	21	1	12	6	2
XIV	2	20	26	3	12	6	1–2
XV	2	20	26	3	12	6	1
XVI	2	20	26	3	12	6	2
XVII (A)	2	22 (11)	35	3	15	6	7
XVII (B)	2	24 (13)	36	3	12	6	3–7
XVIII	2	21	25–26	3	7 *	6	2–8
XIX	2	22 ^ (6 *)	23	3	13	6	2
XX	2	17 *	25–26	3	9	6	6
Further classification of groups of orphan 3FTxs							
21	2	18	25	3	9	6	1
22	2	22	26	3	14	6	1
23	2	22	26	3	12	6	1
24	2	18–19	26–27	3	9	6	2
25	2	18–19	26	3	9	6	2–4
26	2	18	26	3	9	6	2
27	2	22	25	3	13	6	2
28	2	20	26	3	7 *	6	2
29A	2	19	26	3	7 *	6	2
29B	2	19	25	3	7 *	6	8
30	2	20	26	3	9	6	2
31	2	26 (10)	23	3	9	6	2
32	2	18	26	3	9	6	2
33	2	20	25	3	8 *	6	2
34	2	20	25	3	8 *	6	2
35	2	22 ^	23	3	12	6	2
36	2	18	26	3	9	6	2
37	2	22	26	3	12	6	4
38	2	21 ^	22	3	12	6	1–2
39	2	20	27	3	12	6	4
40	2	22 ^	22	3	12	6	2
41	2	22	26	3	12	6	2
42	2	22	26	3	12	6	2
43	2	22	26	3	12	6	2
44	2	20	25	3	8 *	6	2
45	2	20	25	3	8 *	6	6–7
46A	2	20	26	3	12	6	4
46B	2	20	26	3	12	6	4
46C	2	20	26	3	12	6	16 #
47	2	16 *	20 *	1	12	6	2
48	2	19	23	1	12	6	2
49	2	18	26	3	9	6	2
50	2	18	26	3	9	6	2
51	2	20	25	3	8 *	6	2
52	2	20	25	3	8 *	6	8
53	2	22	26	3	12	6	2
54	2	17 *	23	3	9	6	1
55	2	21	23	3	7 *	6	1
56	2	22	26	3	12	6	2
57	2	21	25	3	9	6	2
58	2	22 ^ (6 *)	23	3	11	6	1
59	2	22 ^ (6 *)	23	3	13	6	2
60	2	22 ^ (6 *)	23	3	13	6	1–2
61	2	22 (6 *)	28–29	3	9	6	3
62	2	22 (6 *)	26	3	12	6	4
63	2	22 (6 *)	36	3	10	6	8
64	2	24 ^–25 ^ (10)	24–26	3	9–13	6	1–2
65	2	22–23 (7)	28	5	14	6	2
66	2	22 (6 *)	25	3	13	6	1
67	2	20 (8)	24	3	12	6	2
68	2	19–22 ^	23–26	3	12	6	2
69	2	20	25–26	3	9	6	2
70	2	20	26	3	9	6	2
71	2	20 ^	22	3	9–10	6	2–3
72	2	20	26	3	7 *–9	6	2
73	2	20 ^	22	3	7 *–9	6	2
74	2	20	25	3	7 *–9	6	2–8
75	2	22	25	3	12	6	2
76	2	22	25	3	12	6	1
77	2	21 ^	22	3	12	6	2
78	2	20	27	3	7 *	6	2
79	2	20	23	3	7 *	6	2
80	2	20	23	3	7 *	6	2
81	2	20 ^	21	3	7 *	6	2–3
82	2	20	23	3	7 *	6	2
83	2	22	22	3	12	6	2
84	2	19	26	3	9	6	2–4
85	2	19	27	3	9	6	7
86	2	22	26	3	12	6	1
87	2	22	26	3	12	6	2
88	2	23 (7)	27	5	14–16	6	2
89	2	20–25 ^ (5 *–6 *)	23–26	3	12	6	2–7
90	2	22 ^ (6 *)	22–26	3	11–12	6	1–3
91	2	24 ^ (8)	25	3	12	6	3
92	2	25 ^ (9)	26	3	12	6	8
93	2	25 ^ (9)	23	3	12	6	3
94	2	27 ^ (12)	24	3	9	6	1
95	2	22 (6 *)	26	3	13	6	1
96	2	22 (6 *)	28	3	9	6	2–3
97	2	28 ^ (13)	28	3	9	6	11
98	2	28 ^ (13)	24	3	9	6	3
99	2	24 ^ (6 *)	23–26	3	9	6	3
100	2	22 ^ (6 *)	22	3	12	6	2
101	2	22–24 (6 *–8)	26–27	3	12	6	1–6
102	2	22 (6 *)	26	3	12	6	1
103	2	22 (6 *)	26	3	12	6	4
104	2	22 (6 *)	32	3	12	6	17 #
105	2	22 (6 *)	26	3	12	6	2
106	2	22 (6 *)	26	3	12	6	1
107	2	21 (6 *)	25	3	12	6	2
108	2	21 ^ (6 *)	20 *	1	12	6	1
109	2	22 (6 *)	26	3	12	6	4
110	2	20 (6 *)	24–26	3	12	6	8
111	2	20 (6 *)	26	3	12	6	4
112	2	20 (6 *)	26	3	12	6	2
113	2	24 (10)	27	3	12	6	2
114	2	24 ^ (6 *)	25	3	12	6	1
115	2	22 (6 *)	26	3	12	6	2
N-terminal variants							
116	1	19 (10)	24	3	12	6	3
117	3	26 (10)	27	3	12	6	4
118	3	25 (9)	27	3	12	6	3
119	4	24 ^ (6 *)	21	3	12	6	5
120	4	22 (6 *)	26	3	12	6	4
121	4	22 (6 *)	26	3	12	6	20 #
122	1	23	23	3	12	6	2
Distinct disulfide variants							
123	2	21 (6 *)	23	3	12	0 *	10
124	2	21^ (6 *)	23	3	12	0 *	13 #
125	2	22	25	3	13	0 *	8
126	2	25 (9)	19 *	3	12	6	8
127	2	22^ (6 *)	20 *	3	12	6	7
128	2	26 (9)	27	8	16	6	18 #
129	2	22	26	1	12	6	2
130	2	22	29	3	12	6	3
131	2	21	29	3	12	6	6
132	2	20 ^ (6 *)	20 *–22	3	11	6	2–4
133	2	22	29	3	12	6	1
134	2	18	32	3	12	6	4
135	2	18	26	3	9	6	2
136	2	18	26	3	9	6	2
137	2	18	26	3	9	6	2
138	1	26 ^ (10)	24	3	12	6	4
139	2	?	?	3	12	6	2
140	2	?	?	1	12	6	2
141	2	24 (6 *)	26 ?	3	?	6	2
142	2	20 (6 *)	27 ?	3	?	6	2
143	2	21 (6 *)	23 ?	3	?	6	2
144	2	24 (6 *)	26 ?	3	?	6	2
145	2	19 * (6 *)	24 ?	3	?	6	4
146	2	21 ^–25 ^ (6 *–10)	21–24 ?	3	?	6	1–2
147	2	22 (6 *)	26 ?	3	?	6	1
148	2	22 (6 *)	26 ?	3	?	6	2
149	2	22 (6 *)	26 ?	3	?	6	1–2
150	2	22 (6 *)	26 ?	3	?	6	2
151	2	21 (6 *)	25 ?	3	?	6	2
152	2	21 (6 *)	25 ?	3	?	6	0–1
153	2	19 *	21 ?	3	?	6	2
154	2	22	24 ?	3	?	6	3
155	2	19 *	25	3	7 *-8 ?	6	2
156	2	21	25?	3	?	6	1
157	2	22 (6 ? *)	26	3	12	6	6

* Among the smallest number of amino acid residues in their respective disulfide loop. # Unusually long C-terminal tail. ^ Loop I is almost equal length to or longer than loop II.

## Data Availability

No new data were created or analyzed in this study.
